# Dual regulation of the unfolded protein response by IGF2BP3 during ER stress

**DOI:** 10.1101/gad.353291.125

**Published:** 2026-08-01

**Authors:** Aleksandra S. Anisimova, Sabina Omerbegovic, Milica Mihailovic, Sascha Gratzl, Harald Hornegger, Irmgard Fischer, Gijs A. Versteeg, Stefan L. Ameres, G. Elif Karagöz

**Affiliations:** 1 Max Perutz Labs, Vienna BioCenter, Vienna 1030, Austria;; 2 Vienna BioCenter PhD Program, Doctoral School of the Medical University of Vienna, University of Vienna, Vienna A-1030, Austria;; 3 Medical University of Vienna, Vienna 1030, Austria;; 4 University of Vienna, Vienna 1030, Austria;; 5 Department of Biochemistry and Cell Biology, Center for Molecular Biology, University of Vienna, Vienna 1030, Austria

**Keywords:** IGF2BP3, IRE1, posttranscriptional regulation, RNA-binding proteins, unfolded protein response, endoplasmic reticulum, mRNA decapping complex

## Abstract

Misfolded protein accumulation in the endoplasmic reticulum (ER) perturbs cellular homeostasis, causing pathological ER stress. While a transcriptional response is paramount for the unfolded protein response (UPR), which counters ER protein stress, multiple UPR-linked mRNAs are posttranscriptionally regulated. However, the mechanisms mediating this regulation remain unclear. Here, we reveal specific interactions between the conserved RNA-binding protein IGF2BP3 and transcripts encoding UPR effectors. During ER stress, IGF2BP3 destabilizes many of its target transcripts, including UPR effectors. Mechanistically, ER stress enhances IGF2BP3's association with the mRNA decapping complex and the ER stress sensor RNase IRE1, which correlates with a shift toward mRNA destabilization. Unexpectedly, prolonged depletion of IGF2BP3 inhibits the UPR via decreased transcription of UPR target genes. Together, our findings suggest that IGF2BP3 contributes to proteostasis during ER stress through a dual mechanism: directly promoting mRNA degradation to reduce translation and folding burden and indirectly supporting transcriptional activation of the UPR.

## Abstract (precis)

In this study, Anisimova et al. report that the RNA binding protein IGF2BP3 transcriptionally and poattranscriptionally regulates unfolded protein response (UPR)-linked mRNAs through direct and indirect mechanisms. During ER stress, IGF2BP3 interacts with mRNA decapping and degradation machinery to directly destabilize several target UPR effector transcripts, while also indirectly supporting transcription of UPR target genes.

Protein-folding homeostasis is paramount for robust cell function. Underlining the importance of this homeostasis, various stress-response pathways surveil and react to protein-folding perturbations. In the endoplasmic reticulum (ER), a critical site for the folding of secreted and transmembrane proteins, protein-folding homeostasis is maintained by a conserved signaling cascade known as the “unfolded protein response” (UPR). In metazoans, the UPR is driven by three parallel ER-tethered sensor/transducers: IRE1, PERK, and ATF6. Each sensor monitors the accumulation of misfolded proteins in the ER and takes corrective actions to restore homeostasis.

The UPR increases protein folding and degradation capacity in the ER through transcriptional upregulation of chaperones, foldases, ER-associated degradation (ERAD), and ER-phagy components (Travers et al. 2000; Lee et al. 2003; Sriburi et al. 2004; Bernales et al. 2006; Acosta-Alvear et al. 2007; Bommiasamy et al. 2009; Schuck et al. 2009, 2014; Khaminets et al. 2015; Fumagalli et al. 2016; Grumati et al. 2017). However, if homeostasis is not achieved in a timely manner, the UPR initiates cell death programs that eliminate defective cells for the benefit of the organism (Lin et al. 2007; Lu et al. 2014c). Together with the magnitude and duration of the ER stress, the interplay between the UPR branches sets molecular timers that govern the decision to either repair the damaged cell or eliminate it as a potential threat. These factors heavily depend on cell identity as well as metabolic and cellular state (Lin et al. 2007; Lu et al. 2014c).

While the UPR-driven transcriptional response is essential for maintaining ER homeostasis, cells also utilize a variety of posttranscriptional mechanisms to adapt to ER stress (Harding et al. 1999, 2000; Holcik and Sonenberg 2005; Walter and Ron 2011). The early response to ER stress converges on the shutdown of global translation by the ER-tethered kinase PERK, which reduces global protein synthesis (Harding et al. 1999, 2000; Holcik and Sonenberg 2005; Walter and Ron 2011). During translational shutdown, some translationally silenced mRNAs are protected from turnover, whereas others are degraded (Buchan and Parker 2009; Buchan et al. 2010; Protter and Parker 2016). In parallel, the UPR sensor IRE1 cleaves ER-localized mRNAs via the regulated IRE1-dependent decay of messenger RNAs (RIDD), proposed to decrease the protein folding load in the ER (Hollien and Weissman 2006; Le Thomas et al. 2021). Apart from its role in tuning protein synthesis into the ER, RIDD protects cells from apoptosis via opposing the transcriptional upregulation of the death receptor 5 (DR5) mRNA expression induced by the PERK-ATF4 branch (Lu et al. 2014b). While it is clear that posttranscriptional mechanisms contribute to life-and-death decisions during ER stress, the mechanistic details of how this is achieved have been poorly understood.

RNA-binding proteins (RBPs) mediate posttranscriptional mechanisms that facilitate the adaptation to developmental and metabolic changes or cellular perturbations (Hentze et al. 2018; Gebauer et al. 2021). Using immunoprecipitation coupled to mass spectrometry (IP-MS/MS) analyses of the UPR sensor IRE1, we previously revealed that it associates with the highly conserved RBP IGF2BP3 (insulin-like growth factor 2 mRNA-binding protein 3) in a stress-dependent manner (Acosta-Alvear et al. 2018). IGF2BPs are a conserved family of RBPs that regulate mRNA fate by controlling target mRNA stability, localization, and translation (for review, see Degrauwe et al. 2016). IGF2BPs were proposed to protect mRNAs from degradation by competing with microRNA binding (Jønson et al. 2014; Müller et al. 2018). More recently, IGF2BP family members have been shown to function as readers of m6A and m7G mRNA modifications. While all IGF2BPs guard m6A-modified mRNAs from decay, IGF2BP1 and IGF2BP3 facilitate the degradation of mRNAs harboring m7G modifications (Huang et al. 2018; Liu et al. 2024). Among the three IGF2BP paralogs in mammalian cells, IGF2BP1 and IGF2BP3 are expressed at high levels during early development (Leeds et al. 1997; Mueller-Pillasch et al. 1999; Mori et al. 2001), regulate mRNAs involved in cell growth and migration (Yaniv and Yisraeli 2002; Hüttelmaier et al. 2005; Noubissi et al. 2006; Bell et al. 2013; Jønson et al. 2014; Conway et al. 2016), and are therefore necessary for healthy embryo development. Notably, expression of all IGF2BP paralogs is upregulated in various cancers, and this strongly correlates with poor patient outcomes, enhanced tumor growth, drug resistance, and metastasis (Hansen et al. 2004; Bell et al. 2013; Lederer et al. 2014).

Several studies show that during proteotoxic stress induced by heat shock or arsenite treatment, IGF2BPs promote mRNA stability by preventing mRNA degradation. Moreover, they facilitate target mRNA translation once the stress is relieved (Stöhr et al. 2006; Weidensdorfer et al. 2009; Huang et al. 2018). Given these data, IGF2BPs may mediate posttranscriptional regulation in response to organelle-specific stress. However, whether IGF2BP3 responds to ER stress and contributes to restoring ER homeostasis remains unclear.

In this study, we assessed the role of IGF2BP3 in regulating UPR-associated transcripts. Our data show that the stress-dependent association of IGF2BP3 with IRE1 is conserved across cell lines, revealing that IGF2BP3 binds to prominent UPR effector transcripts. Using genome-wide transcriptome and mRNA stability analyses, we demonstrate that IGF2BP3 regulates the UPR at both the posttranscriptional and transcriptional levels, thereby forming a unique gene regulatory network that modulates the UPR.

## Results

### IGF2BP3 associates with IRE1 during ER stress

To decipher posttranscriptional mechanisms regulating mRNA fate during ER stress, we focused on the primary posttranscriptional regulator, the ER-tethered RNase IRE1, which binds to and cleaves the ER-bound mRNAs during ER stress. To this end, we characterized the interactome of the ER-tethered RNase IRE1, a key posttranscriptional regulator that binds and cleaves ER-localized mRNAs during ER stress. In a previous work, we mapped the IRE1 interactome by IP-MS/MS in HEK293T and multiple myeloma cells and identified select RBPs associated with IRE1 (Acosta-Alvear et al. 2018). We hypothesized that RBPs that interact with IRE1 during ER stress regulate the stability of UPR-relevant mRNAs associated with IRE1. Among those RBPs, IGF2BP3 stood out, as it was also recovered in IRE1 IP-MS/MS under denaturing conditions after RNA-protein cross-linking, suggesting a robust association with IRE1 and RNA-containing complexes (Supplemental Fig. S1A).

**Figure 1.**
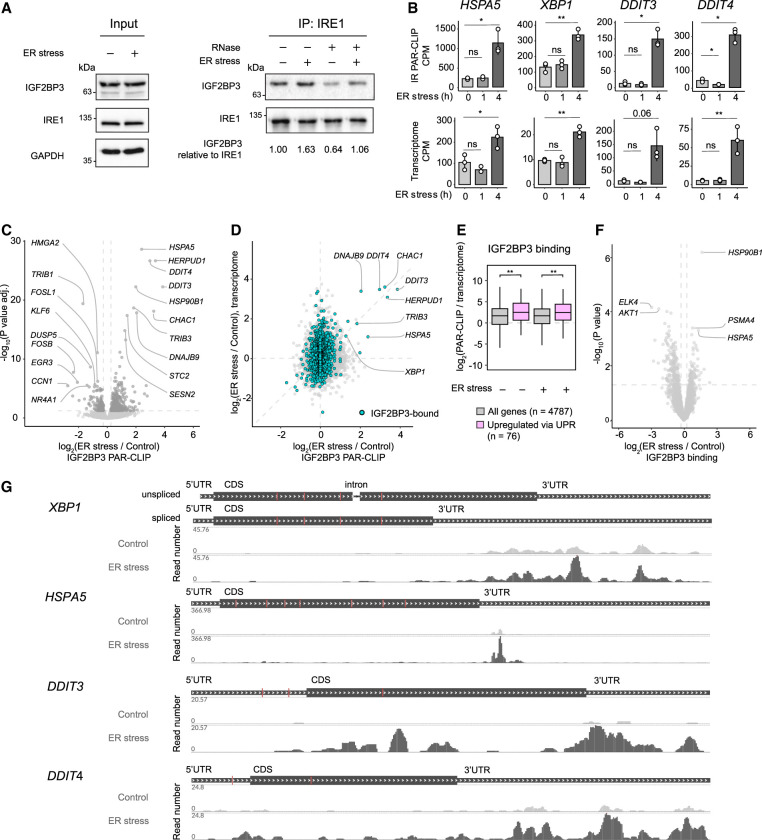
IGF2BP3 interacts with the UPR target mRNAs. (*A*) Western blot of IGF2BP3 showing its association with IRE1 after immunoprecipitation of endogenous IRE1 from HCT116 WT cells treated with ER stress-inducing drug TM at 5 µg/mL for 4 h with or without RNase in the washes. (*B*) Normalized read count numbers (CPM) for IGF2BP3-bound reads (IR-PAR-CLIP) and total transcript levels (QuantSeq) of selected UPR target genes upon ER stress induction with TM at 5 µg/mL for 1 and 4 h. Data are the mean ± SD of *n* = 3 biological replicates. *P*-values were calculated by unpaired two-sided Student's *t*-test. (*C*) Volcano plot showing changes in IGF2BP3 IR-PAR-CLIP CPM upon ER stress treatment. *P*-values were calculated by edgeR glmQLFTest. (*D*) Scatter plot comparing changes in IGF2BP3 IR-PAR-CLIP read counts with the total transcriptome changes (QuantSeq) upon ER stress treatment. (*E*) Box plot showing changes in IGF2BP3 binding (IR-PAR-CLIP CPM/QuantSeq CPM) to all genes and UPR upregulated genes (upregulated more than twofold in ER stress than in control, adjusted *P*-value of <0.05, total RNA-seq) upon ER stress and control conditions. *P*-values were calculated by two-sided Wilcoxon test. (*F*) Volcano plot showing changes in IGF2BP3 binding (IR-PAR-CLIP CPM/QuantSeq CPM) upon ER stress and control conditions. IGF2BP3-bound transcripts are shown. *n* = 3 biological replicates. *P*-values were calculated by edgeR glmQLFTest. (*G*) Representative IGF2BP3 IR-PAR-CLIP coverage examples. Where not indicated otherwise, ER stress was induced with TM at 5 µg/mL for 4 h. For IR-PAR-CLIP and transcriptome experiments *n* = 3 biological replicates. (*) *P* < 0.05, (**) *P* < 0.01.

To test whether the ER stress-induced increase in IGF2BP3 association with IRE1 extended across different cell types, we performed endogenous IRE1 IPs followed by Western blot analysis in HCT116 cells. IGF2BP3's interaction with IRE1 increased 1.6-fold after 4 h of ER stress compared to unstressed controls (Fig. 1A). Consistent with this result, we also recovered IRE1 using reciprocal IGF2BP3 pull-downs in HEK293T cells in an ER stress-dependent manner (Supplemental Fig. S1B). To address whether IGF2BP3 and IRE1 interaction is mediated by their binding to RNA, we performed IRE1 IPs in the presence of RNase. We found that the interaction between IRE1 and IGF2BP3 was partially sensitive to RNase treatment, indicating that both RNA-protein and protein–protein interactions contribute to their association (Fig. 1A). From these results, we concluded that during ER stress, IRE1 and IGF2BP3 exist in RNA-protein complexes. The fact that these complexes were identified across multiple cell types suggests their potential importance for general cellular biology.

### IGF2BP3 interacts with UPR target mRNAs

The mRNAs interacting with IGF2BP3 under steady-state conditions have been well established (Hafner et al. 2010; Conway et al. 2016; Anisimova and Karagöz 2023), but it is unclear whether proteotoxic stress alters IGF2BP3's mRNA-binding landscape. To address this, we defined IGF2BP3-bound mRNAs during ER stress in HCT116 cells using infrared photoactivatable ribonucleoside-enhanced cross-linking and immunoprecipitation (IR-PAR-CLIP) (Anisimova and Karagöz 2023). Due to extensive RNase cleavage and the presence of T-to-C (T-C) transitions, which mark the cross-link position, this approach allows nucleotide-resolution detection of RBP-interaction sites in target RNAs (Hafner et al. 2010). IGF2BP3 IR-PAR-CLIP was performed under homeostatic conditions, during early response (1 h), and at peak response (4 h) time points after UPR induction by TM. As UPR target genes are transcriptionally upregulated under these conditions, we performed parallel total transcriptome sequencing to account for changes in transcript levels.

On average, IGF2BP3 IR-PAR-CLIP produced 45-times more unique deduplicated reads relative to the IgG controls, with ∼50% of the reads containing T-C transitions (Supplemental Fig. S1C). To identify IGF2BP3-bound transcripts, we performed T-C-aware peak calling with PARalyzer (Corcoran et al. 2011). Transcripts containing at least one cluster of IGF2BP3-cross-linked reads with more than 25 gene counts per million (CPM) and over 50% T-C conversions per read were considered IGF2BP3-bound. After correcting for transcript levels (IR-PAR-CLIP CPM > transcriptome CPM), IR-PAR-CLIP identified 2313 IGF2BP3-bound transcripts under homeostatic conditions (Supplemental Fig. S1D; Supplemental Table S1). A short ER stress induction (1 h) did not significantly alter either the levels of the hallmark UPR target transcripts or IGF2BP3 binding; however, a 4 h treatment led to strong UPR induction (Fig. 1B). Under these conditions, IGF2BP3 interacted with a transcript set largely overlapping that observed under control conditions, with 157 additional transcripts interacting exclusively during ER stress (Supplemental Fig. S1D). These transcripts were enriched in those involved in ER stress response and proteasomal degradation (Supplemental Fig. S1E). We further defined a list of 2470 IGF2BP3-bound transcripts present in either control or ER stress (4 h) conditions (Supplemental Table S1). Gene ontology analyses of those mRNAs revealed that regulation of transcription, protein degradation, differentiation, intracellular communication, and response to ER stress were the top-enriched biological processes (Supplemental Fig. S1F).

In agreement with the published data, our IGF2BP3 IR-PAR-CLIP analyses showed that under homeostatic conditions, IGF2BP3 binds to most of its target mRNAs through their 3′ UTRs (Supplemental Fig. S1G; Hafner et al. 2010; Anisimova and Karagöz 2023). While IGF2BP3's binding preference for 3′ UTRs was not affected by ER stress, the IGF2BP3-binding motifs showed distinct differences (Supplemental Fig. S2A). The top five 5 nt long motifs enriched in IGF2BP3-bound IR-PAR-CLIP reads included those with CA-rich sequences common among IGF2BP3 binding motifs (Conway et al. 2016; Schneider et al. 2019). Three motifs (UCCAG, AGCCU, and UGCCA) were common for both conditions. In contrast, upon stress, novel ACUGU and ACCUG motifs were highly enriched in comparison to a canonical CAUU-containing motif (Hafner et al. 2010). Fluorescence anisotropy experiments demonstrated that purified IGF2BP3 binds RNAs containing the ACUGU motif, which is enriched under ER stress, with an affinity comparable to that observed for the canonical UCCAG and CAUUU motifs (*K*_d_ ≈ 400 nM). In contrast, we only detected low-affinity binding to a negative control RNA (Supplemental Fig. S2B; Supplemental Table S1). These results indicated that IGF2BP3 associates with novel sequences during ER stress.

Next, we tested whether ER stress alters the pool of transcripts associated with IGF2BP3. Upon ER stress induction, the number of IGF2BP3-bound reads derived from UPR-induced genes increased. These UPR targets included genes encoding for the master transcription factors XBP1, ATF4, and CHOP (*DDIT3*) and the major chaperones BiP (*HSPA5*) and DNAJB9 (Fig. 1C). At the same time, some canonical targets of IGF2BP3, such as *HMGA2* mRNA (Jønson et al. 2014), were depleted from the IGF2BP3-bound transcript pool (Fig. 1C). To assess the binding of IGF2BP3 to the UPR target transcripts we defined 228 genes that were upregulated at least twofold upon ER stress induction as “UPR-induced.” To account for changes in mRNA levels, we next compared ER stress-induced changes in transcript levels with changes in IGF2BP3-bound transcripts (Fig. 1D) and calculated an “IGF2BP3 binding” score by normalizing the IR-PAR-CLIP reads to transcript levels (Fig. 1E,F). These analyses revealed that IGF2BP3 binds UPR-induced transcripts to a similar extent in both homeostatic and ER stress conditions, suggesting that for most genes the observed increase in IGF2BP3-bound reads results from increased transcription of UPR target genes during ER stress. However, while for multiple UPR targets IGF2BP3-bound reads increased linearly with increased expression, some transcripts such as *HSPA5* and *HSP90B1*, showed preferential IGF2BP3 binding during ER stress (Fig. 1F,G; Supplemental Fig. S2C). Additionally, we identified several novel IGF2BP3 targets, such as *DDIT3*, that were expressed only during ER stress (Fig. 1B,G).

IGF2BP3 is a reader of m^6^A and internal m^7^G-modified RNAs (Huang et al. 2018; Liu et al. 2024). As mRNA methylation is regulated under proteotoxic stress (Zhou et al. 2015; Wei et al. 2021), stress-induced changes in methylation could alter IGF2BP3 target association. We therefore tested whether these motifs are more frequent in IGF2BP3-bound reads under ER stress than under unstressed conditions. m^6^A modifications predominantly occur on consensus sequence motifs and are enriched in 5′-DRA*CH-3′ sequences (DRACH, D = A, G or U; H = A, C or U) (Linder et al. 2015). Analysis of the consensus DRACH motif in IGF2BP3-binding sites showed a stress-dependent increase in the enrichment of this motif (Supplemental Fig. S2D). Similarly, the consensus motifs for internal m^7^G deposition derived from recent transcriptome-wide mapping approaches (Malbec et al. 2019; Zhao et al. 2023) showed a stress-dependent increase in the occurrence of these motifs (GANGAN) in IGF2BP3-bound RNAs, indicating that the deposition of these modifications might contribute to IGF2BP3's RNA selectivity during ER stress (Supplemental Fig. S2D). In line with these findings, the strongest IGF2BP3 binding peaks at the *HSPA5* 3′ UTR correspond to experimentally confirmed m^6^A (see the Materials and Methods; Liang et al. 2024), and m^7^G (Malbec et al. 2019) deposition sites (Supplemental Fig. S2E). These analyses suggest that alterations in these mRNA modifications may influence IGF2BP3 binding to its target transcripts during ER stress.

Supporting our IP-MS/MS data, IGF2BP3 was bound to one-third of the transcripts interacting with IRE1 during ER stress (Supplemental Fig. S2F). The IGF2BP3-binding sites did not overlap with IRE1 RIDD cleavage sites on most of the common IRE1 and IGF2BP3-bound mRNAs. Those analyses suggested that IGF2BP3 binding does not compete with IRE1 (Supplemental Fig. S2G). To sum up, we found that IGF2BP3 interacts with transcripts encoding UPR target genes during ER stress, identifying IGF2BP3 as a potential posttranscriptional regulator of the UPR.

### IGF2BP3 depletion dampens UPR signaling

**Figure 2.**
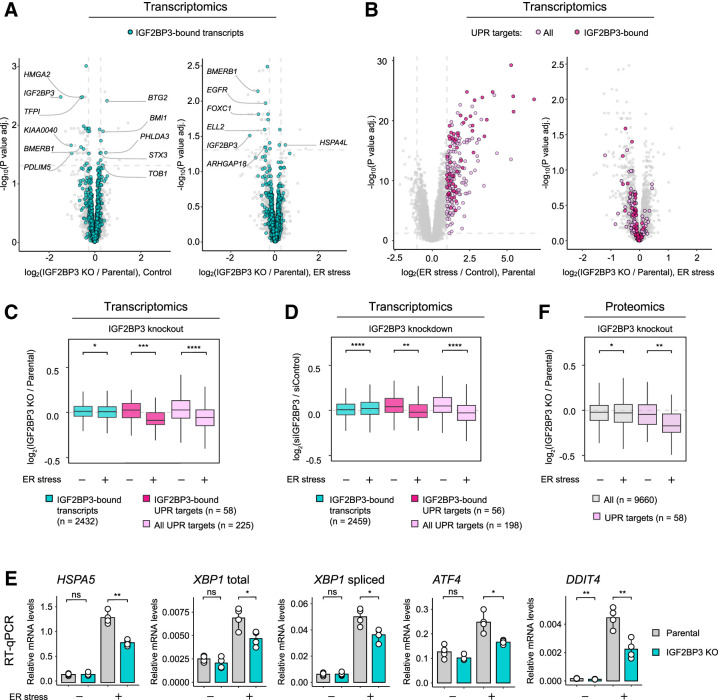
IGF2BP3 depletion dampens the unfolded protein response. (*A*) Volcano plot of transcriptome (RNA-seq) changes upon IGF2BP3 KO in control (*left* panel) and ER stress conditions (*right* panel). *P*-values were calculated by edgeR glmQLFTest. (*B*) Volcano plot of transcriptome (RNA-seq) changes upon ER stress treatment showing the selection of UPR targets (*left* panel), and volcano plot upon IGF2BP3 KO in ER stress conditions with highlighted UPR targets (upregulated more than twofold in ER stress than in control, adjusted *P*-value of <0.05, total RNA-seq) and IGF2BP3-bound UPR targets (*right* panel). *P*-values were calculated by edgeR glmQLFTest. (*C*) Box plot showing changes in transcript levels upon IGF2BP3 KO for IGF2BP3-bound transcripts, UPR targets, and IGF2BP3-bound UPR targets. *P*-values were calculated by two-sided Wilcoxon test. (*D*) Box plot showing changes in transcript levels upon siRNA-mediated IGF2BP3 for IGF2BP3-bound transcripts, UPR targets, and IGF2BP3-bound UPR targets. *n* = 3 biological replicates. *P*-values were calculated by two-sided Wilcoxon test. (*E*) RT-qPCR analyses of relative mRNA levels for UPR targets (*HSPA5* and *XBP1* spliced and total, and *ATF4* and *DDIT4*) in IGF2BP3 KO and parental cells treated with 100 ng/mL TM for 7 h. *n* = 4 biological replicates. *P*-values were calculated by unpaired two-sided Student's *t*-test. (*F*) Box plot showing changes in protein levels (total proteome mass spectrometry) upon IGF2BP3 KO for all identified proteins and UPR targets (upregulated more than 25% in ER stress than in control, adjusted *P*-value of <0.05, total proteome). ER stress was induced with 100 ng/mL TM for 7 h. *P*-values were calculated by two-sided Wilcoxon test. *n* = 4 biological replicates. If not indicated otherwise, ER stress was induced with TM at 5 µg/mL for 4 h. For the transcriptome analysis IGF2BP3 KO cell three clones (in duplicates) and parental cells in quadruplicate were used. In RT-qPCR analyses, mRNA levels were normalized to *RPL6*. (*) *P* < 0.05, (**) *P* < 0.01, (***) *P* < 0.001, and (****) *P* < 0.0001.

Based on our findings above, we hypothesized that IGF2BP3 might posttranscriptionally regulate UPR-induced mRNAs. We therefore set out to identify transcripts regulated by IGF2BP3. To this end, IGF2BP3 was depleted in colon carcinoma HCT116 cells by CRISPR/Cas9-mediated knockout (Supplemental Fig. S3A), and transcriptome analyses were performed under homeostatic and ER-stress conditions (Supplemental Table S2). IGF2BP3 knockout resulted in moderate changes in the total transcriptome (Fig. 2A). Specifically, it led to the downregulation of genes involved in the regulation of transcription, cell proliferation and migration, development, and response to growth factors, consistent with previous reports (Supplemental Fig. S3B; Lederer et al. 2014; Ennajdaoui et al. 2016; Mizutani et al. 2016; Anisimova and Karagöz 2023). To address the effect of IGF2BP3 depletion on the cellular response to ER stress, we defined 288 UPR target transcripts as those upregulated by more than twofold upon ER stress, including those bound by IGF2BP3 (58 transcripts) (Fig. 2B, left panel) and followed their levels upon IGF2BP3 depletion (Fig. 2B, right panel). Transcriptome analysis showed that levels of UPR target transcripts were lower in IGF2BP3 knockouts compared to the parental cells under ER stress (Fig. 2B,C). While IGF2BP3 depletion had only a moderate impact on individual UPR effector transcripts (Fig. 2B), changes were evident when analyzed for the whole group (Fig. 2C). In contrast, the levels of other IGF2BP3-bound transcripts were less affected by IGF2BP3 CRISPR/Cas9-mediated knockout (Fig. 2A,C). Thus, IGF2BP3 specifically impacts UPR target transcripts during ER stress (Fig. 2B,C).

To account for cell adaptation to long-term IGF2BP3 depletion and to bypass clonal selection, we used siRNA-based depletion as an orthogonal approach to validate our findings (Supplemental Table S2). Using an siRNA pool targeting IGF2BP3, we obtained 75% depletion within 48 h in HCT116 cells (Supplemental Fig. S3C,D). The siRNA knockdown experiments confirmed the data obtained by the CRISPR/Cas9-based IGF2BP3 knockouts, indicating that depletion of IGF2BP3 dampens UPR signaling (Fig. 2D). Reverse transcription quantitative PCR (RT-qPCR) analyses of IGF2BP3-interacting transcripts confirmed these results at milder, closer to physiological ER stress conditions, showing that IGF2BP3 knockout leads to a decrease in the levels of major UPR targets downstream from PERK (*ATF4* and *DDIT4*), IRE1 (*XBP1* total and spliced), and ATF6 (*HSPA5*) (Fig. 2E). Intriguingly, our analyses showed that during ER stress, depletion of IGF2BP3 not only reduced the levels of IGF2BP3-bound UPR targets but also affected those that are not its direct interactors (Fig. 2B–D). The data suggested that IGF2BP3 depletion leads to the downregulation of the entire pathway, possibly through secondary, indirect mechanisms. We confirmed these findings using global proteomic analysis of wild-type cells and IGF2BP3 knockouts, demonstrating that IGF2BP3 depletion decreased the protein abundance of UPR targets during ER stress (Fig. 2F; Supplemental Fig. S3E; Supplemental Table S3). Altogether, our data showed that IGF2BP3 depletion tunes down UPR.

### IGF2BP3 shapes the UPR through transcriptional feedback loops

As mentioned above, our genome-wide approaches revealed that, in addition to IGF2BP3-bound transcripts, the levels of UPR targets not directly bound to IGF2BP3 also decreased upon IGF2BP3 depletion, suggesting a secondary, indirect effect (Fig. 2B–D,F). Because IGF2BP3 controls the stability of several mRNAs involved in transcriptional regulation, we hypothesized that the indirect effects observed upon IGF2BP3 depletion result from transcriptional reprogramming.

**Figure 3.**
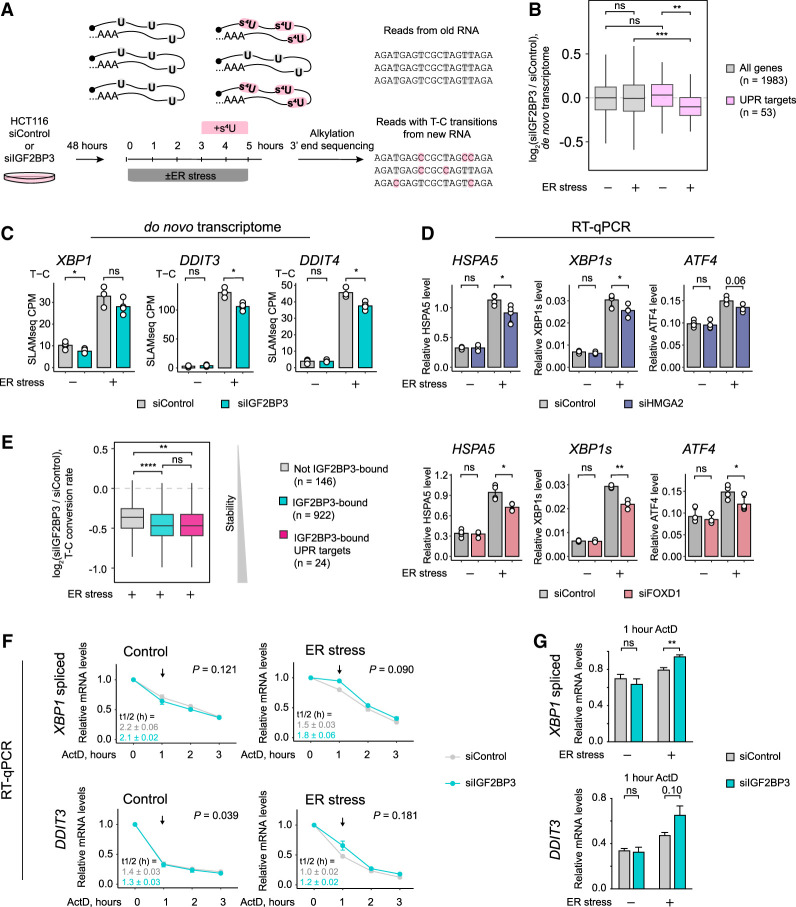
IGF2BP3 shapes the UPR through transcriptional feedback loops. (*A*) Design of the thiol (SH)-linked alkylation for the metabolic sequencing of RNA (SLAMseq) experiment. IGF2BP3 is depleted for 48 h using siRNA. Prior to collection cells were treated with TM at 5 µg/mL or with DMSO as a control for 5 h. In the last 2 h 100 µM 4-thiouridine (s^4^U) was added to the cells to label the newly synthesized mRNA. (*B*) Box plot showing changes in de novo transcripts levels of UPR targets (transcribed during 2 h s^4^U pulse) T-C (SLAMseq T-C CPM) upon siRNA-mediated depletion of IGF2BP3. *P*-values were calculated by two-sided Wilcoxon test. (*C*) SLAMseq de novo (T-C) CPM values for selected UPR target genes (*XBP1*, *DDIT4*, and *DDIT3*). Data are the mean ± SD of *n* = 4 biological replicates. *P*-values were calculated by unpaired two-sided Student's *t*-test. (*D*) RT-qPCR of *HSPA5*, *XBP1* spliced, and *ATF4* in unstressed cells and cells treated with TM at 100 ng/mL for 8 h upon siRNA-mediated depletion of HMGA2 (*top* panel) and FOXD1 (*bottom* panel). Data are the mean ± SD of *n* = 4 biological replicates. *P*-values were calculated by unpaired two-sided Student's *t*-test. (*E*) Box plot showing changes in estimated transcript stability upon ER stress following siRNA-mediated IGF2BP3 depletion for IGF2BP3-bound transcripts, IGF2BP3-bound UPR targets, and non-IGF2BP3 bound transcripts (IR-PAR-CLIP CPM/QuantSeq CPM < 0.1). The changes in transcript stability were assessed by analyzing the differences in average T-C conversion rates per gene following IGF2BP3 depletion. T-C conversion rate values indicate the relative abundance of both preexisting and newly synthesized mRNA for a given gene, with a decrease in these values suggesting increased stabilization. *P*-values were calculated by two-sided Wilcoxon test. (*F*) RT-qPCR analyses of degradation rates of the UPR targets *XBP1* (spliced) and *DDIT3* upon siRNA-mediated depletion of IGF2BP3. HCT116 cells were treated with TM at 250 ng/mL for 4 h and the transcription was blocked with 5 µg/mL ActD for indicated time points. Data are the mean ± SE of *n* = 4 biological replicates. *P*-values were calculated by paired two-sided Student's *t*-test. (*G*) Bar plots showing results of RT-qPCR analyses of *XBP1* (spliced) and *DDIT3* total mRNA levels in unstressed cells and cells treated with TM at 250 ng/mL for 4 h. Arrows indicate the 1 h ActD treatment time point selected for box plot representation in *E*. *P*-values were calculated by unpaired two-sided Student's *t*-test. For the SLAMseq experiments *n* = 4 biological replicates. In RT-qPCR analyses, mRNA levels were normalized to *RPL6*. (*) *P* < 0.05, (**) *P* < 0.01, (***) *P* < 0.001, (****) *P* < 0.0001.

To uncover the primary mechanism of IGF2BP3-mediated regulation during ER stress, we aimed to uncouple transcriptional reprogramming from posttranscriptional regulation. To test whether transcriptional reprogramming contributes to the changes in the transcript levels upon IGF2BP3 depletion, we used thiol (SH)-linked alkylation for the metabolic sequencing of RNA (SLAMseq) method (Fig. 3A). In SLAMseq, nascent transcripts are metabolically labeled, allowing assessment of de novo RNA synthesis (Herzog et al. 2017). We performed 2 h of pulse labeling in HCT116 cells with s^4^U, followed by total RNA extraction, alkylation, and mRNA 3′-end library preparation under homeostatic conditions and after 5 h of ER stress (with s^4^U added for the last 2 h) (Supplemental Table S4). First, we followed changes in transcript levels synthesized during the 2 h of the s^4^U pulse (de novo transcriptome and T-C reads). SLAMseq analyses showed that siRNA-mediated depletion of IGF2BP3 decreased transcription of the UPR target genes (Fig. 3B,C), indicating that IGF2BP3 indirectly contributes to transcriptional reprogramming, potentially by affecting the expression of one or more transcriptional regulators. Indeed, several mRNAs encoding for transcription factors were downregulated upon IGF2BP3 depletion (Supplemental Fig. S4A).

To dissect this transcriptional circuitry, we focused on two top candidate regulators whose mRNA levels decreased strongly upon IGF2BP3 depletion. The canonical IGF2BP3 target *HMGA2* (Supplemental Fig. S4B; Jønson et al. 2014), which is a nonhistone chromatin factor (Pfannkuche et al. 2009), and a *forkhead* box transcription factor FOXD1 (Supplemental Fig. S4C; Hatini et al. 1996; Quintero-Ronderos and Laissue 2018). siRNA-mediated knockdown of either HMGA2 or FOXD1 significantly reduced levels of the UPR target transcripts specifically under ER stress, indicating a functional contribution to the UPR (Fig. 3D; Supplemental Fig. S4D,E). In contrast, depleting these factors did not affect the control transcripts *UBC* and *EEF1A1*, which are transcribed at high rates but not ER stress-regulated, supporting the selective role of HMGA2 and FOXD1 in UPR regulation (Supplemental Fig. S4F,G). Notably, HMGA2 knockdown also reduced *FOXD1* mRNA levels (Supplemental Fig. S4H), suggesting that HMGA2 acts upstream in this transcriptional program. In line with this model, IR-PAR-CLIP did not detect IGF2BP3 binding to *FOXD1* mRNA (Supplemental Table S1). Moreover, depletion of either IGF2BP3 or HMGA2 produced similar effects on the UPR target transcripts, and codepletion did not show an additive effect (Supplemental Fig. S4I). These data suggest that HMGA2 is the primary driver of the transcriptional programming downstream from IGF2BP3.

To systematically assess the contribution of posttranscriptional regulation to the levels of UPR target transcripts, we calculated changes in mRNA stability by comparing per-gene average T-C conversion rates following IGF2BP3 depletion. In SLAM-seq, T-C conversions mark newly synthesized (s^4^U-labeled) RNA. The T-C conversion rate represents the ratio of T-C conversions to the total T coverage per gene (see the Materials and Methods) and therefore reflects the relative contribution of newly synthesized versus preexisting mRNA. A decrease in the T-C conversion rate indicates stabilization, whereas an increase indicates destabilization. Surprisingly, our analyses showed that IGF2BP3 depletion increased the stability of IGF2BP3-bound transcripts, including those encoding for the UPR target genes, particularly during ER stress (Fig. 3E; Supplemental Fig. S4J). These data indicated that IGF2BP3-driven posttranscriptional and transcriptional mechanisms control UPR transcript levels in opposing directions.

Consistent with IGF2BP3 adopting a more destabilizing function under stress, when transcription was inhibited by actinomycin D (ActD) treatment, IGF2BP3 depletion increased the levels of *XBP1* (spliced and total) mRNAs during ER stress (Fig. 3F,G; Supplemental Fig. S4K). A similar trend was observed for *DDIT3* (CHOP) (Fig. 3F,G). Notably, this destabilization effect was specific to ER stress and was not observed for a nontarget control transcript, *UBC* (Supplemental Fig. S4L). These data suggested that IGF2BP3 regulates the UPR in two ways: (1) It binds to and destabilizes its target mRNAs during ER stress. (2) It indirectly upregulates the transcription of the UPR target genes. This transcriptional regulatory mechanism supports differential regulation of UPR target transcripts by IGF2BP3 during ER stress. The transcriptional response is more potent, and this way, IGF2BP3 facilitates the UPR. This model explains how IGF2BP3 depletion leads to decreased levels of the UPR target transcripts, while its depletion increases the stability of its posttranscriptional targets. The IGF2BP3-driven dual regulatory mechanism might allow cells to reduce the protein-folding burden while ensuring they elicit a potent UPR during ER stress.

### IGF2BP3 facilitates mRNA degradation during ER stress

Our transcriptomic and SLAMseq analyses showed that siRNA-mediated IGF2BP3 depletion led to both stabilization and destabilization of IGF2BP3-bound transcripts under homeostatic conditions. Most top-regulated transcripts encoded proteins involved in cell proliferation and motility (e.g., *HMGA2*, *CARM1*, *TRIB1*, and *NACC2*) (An et al. 2004; Frietze et al. 2008; Xuan et al. 2013; Singh et al. 2023). Notably, several of these target mRNAs encoded transcription factors (*ZNF385A*), regulators of gene expression (*HMGA2*, *CARM1*, and *NACC2*), or RNA metabolism (*AGO2* and *ZFP36L1*), underlining that IGF2BP3-mediated posttranscriptional regulation impinges on potent gene regulatory networks to remodel the transcriptome.

**Figure 4.**
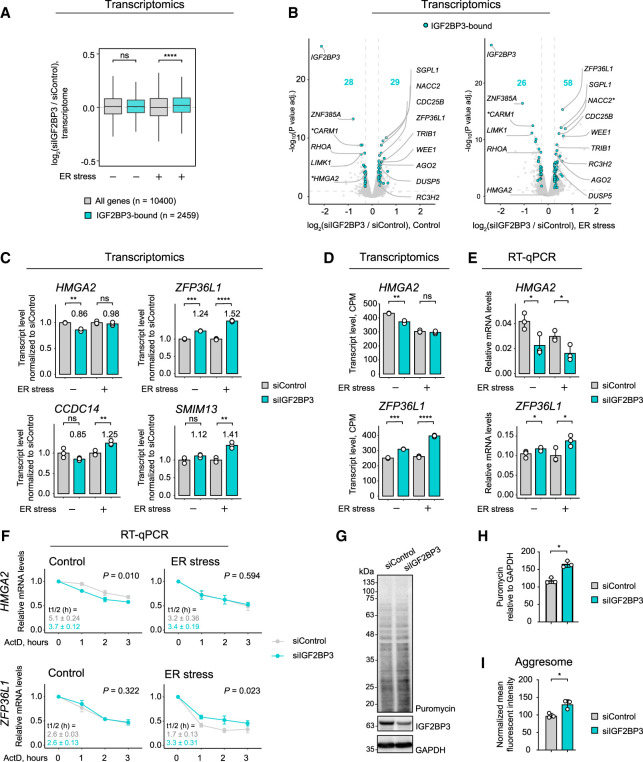
IGF2BP3 facilitates the degradation of its target mRNAs during ER stress. (*A*) Box plot showing changes in total transcript levels (RNA-seq) upon siRNA-mediated depletion of IGF2BP3 for all genes and IGF2BP3-bound transcripts in control and ER stress conditions. (*B*) Volcano plots of total transcriptome changes (RNA-seq) upon siRNA-mediated IGF2BP3 depletion with regulated genes highlighted (ΔRNA-seq >20% and adjusted *P*-value of <0.05). Asterisks (*) mark the genes encoding transcriptional regulators. (*C*) Bar plots showing total RNA-seq CPM values normalized to siControl conditions for selected genes regulated by IGF2BP3. Data are the mean ± SD of *n* = 3 biological replicates. *P*-values were calculated by unpaired two-sided Student's *t*-test. Numbers indicate normalized mean data. (*D*) Bar plots showing total RNA-seq CPM values for *HMGA2* and *ZFP36L1*. Data are the mean ± SD of *n* = 3 biological replicates. *P*-values were calculated by unpaired two-sided Student's *t*-test. (*E*) Bar plots showing results of RT-qPCR analyses of *HMGA2* and *ZFP36L1* in unstressed cells and cells treated with TM at 5 µg/mL for 6 h upon siRNA-mediated depletion of IGF2BP3. Data are the mean ± SD of *n* = 3 biological replicates. *P*-values were calculated by unpaired two-sided Student's *t*-test. (*F*) RT-qPCR analyses of degradation rates for *HMGA2* and *ZFP36L1* upon siRNA-mediated depletion of IGF2BP3. HCT116 cells were treated with TM at 5 µg/mL for 3 h and the transcription was blocked with ActD for indicated time points. Data are the mean ± SE of *n* = 3 biological replicates. *P*-values were calculated by paired two-sided Student's *t*-test. (*G*) Western blot showing global protein synthesis (30 min puromycin pulse) upon siRNA-mediated depletion of IGF2BP3 in ER stress conditions. (*H*) Quantification of *G*. Data are the mean ± SD of *n* = 3 biological replicates. *P*-values were calculated by unpaired two-sided Student's *t*-test. (*I*) Flow cytometry analysis of aggresome formation using the ProteoStat dye upon siRNA-mediated depletion of IGF2BP3 in ER stress conditions. Data are the mean ± SD of *n* = 3 biological replicates. *P*-values were calculated by unpaired two-sided Student's *t*-test. For the transcriptome analyses *n* = 3 biological replicates. In RT-qPCR analyses, mRNA levels were normalized to *RPL6*. ER stress was induced with 5 µg/mL TM for 4 h. (*) *P* < 0.05, (**) *P* < 0.01, (***) *P* < 0.001, (****) *P* < 0.0001.

The SLAM-seq analyses also revealed that, in contrast to homeostatic conditions, IGF2BP3 binding is associated with destabilization of its target mRNAs during ER stress (Fig. 3E). The observed increase in IGF2BP3-mediated destabilization upon ER stress was specific for IGF2BP3-bound transcripts (Fig. 3E) and was also evident at the level of total mRNA abundance (Fig. 4A). In line with these analyses, IGF2BP3 destabilized a higher number of transcripts during ER stress compared to homeostatic conditions (Fig. 4B,C). Together, these findings suggest that ER stress shifts IGF2BP3 function toward destabilizing its target transcripts.

Our data confirmed that IGF2BP3 stabilizes *HMGA2* mRNA (Jønson et al. 2014), whereas it destabilizes *ZFP36L1* mRNA (Ennajdaoui et al. 2016), as reported in earlier studies (Fig. 4B,D,E). In line with the published work, depletion of IGF2BP3 resulted in lower levels of *HMGA2* mRNA under homeostatic conditions, supporting the notion that IGF2BP3 binding protects this transcript from decay (Fig. 4B–D). The SLAMseq analyses further showed that the regulation is achieved at the posttranscriptional level, as IGF2BP3 depletion did not result in decrease of *HMGA2* transcription (Supplemental Fig. S5A). Using RT-qPCR analyses, we confirmed that IGF2BP3 did not stabilize *HMGA2* mRNA as efficiently during ER stress as under homeostatic conditions (Fig. 4E). These results were corroborated by actinomycin D chase experiments, which showed that IGF2BP3 depletion decreased the half-life of *HMGA2* mRNA under homeostatic conditions while not impacting it during ER stress (Fig. 4F).

Consistent with our data suggesting that IGF2BP3 switches to a degradative function during ER stress, we observed more pronounced destabilization of a subset of transcripts that IGF2BP3 also destabilizes under homeostatic conditions (Fig. 4B,C; Supplemental Fig. S5A). Both the transcriptomics (Fig. 4B–D; Supplemental Fig. S5A) and RT-qPCR data (Fig. 4E) showed that IGF2BP3 depletion increased the levels of a well-described target, *ZFP36L1* mRNA under homeostatic conditions, supporting earlier work (Ennajdaoui et al. 2016) that proposed IGF2BP3 facilitates *ZFP36L1* mRNA degradation. This effect was more pronounced during ER stress (Fig. 4C–E), with *ZFP36L1* mRNA showing an increased half-life upon IGF2BP3 depletion under stress conditions in actinomycin D chase experiments (Fig. 4F). These analyses indicated that ER stress modulates IGF2BP3 function, shifting the functional consequences of IGF2BP3 binding toward mRNA degradation.

We next asked whether IGF2BP3-mediated mRNA degradation reduces translational burden during ER stress. To measure global translational output, we performed puromycin incorporation assays in wild-type and IGF2BP3-depleted cells under ER stress. IGF2BP3 knockdown resulted in increased puromycin incorporation, indicating elevated translation (Fig. 4G,H). Importantly, we found that depletion of IGF2BP3 under those conditions led to increased protein aggregation (Fig. 4I). These results reveal the importance of IGF2BP3-mediated posttranscriptional regulation in maintaining a balance between translation and protein folding during the ER stress. To summarize, these findings suggest that IGF2BP3-dependent mRNA degradation helps limit translational burden during ER stress.

### ER stress promotes the association of IGF2BP3 with the mRNA decapping complex

The association of IGF2BP3 with the mRNA degradation machinery was previously proposed to regulate a subset of its target mRNAs (Ennajdaoui et al. 2016; Mizutani et al. 2016; Latifkar et al. 2022). Therefore, we employed proteomics to investigate whether changes in the IGF2BP3 interactome explain the increase in IGF2BP3-mediated degradation during ER stress. To this end, we immunoprecipitated IGF2BP3 from HCT116 cells (Supplemental Table S5). The proteomics analyses showed that IGF2BP3 co-IP recovered proteins involved in almost all distinct steps of mRNA metabolism including transcription (RNA Pol II subunits, transcription factors), mRNA splicing (spliceosome components, SR proteins), export (THO complex subunits, MAGOH, RBM8A) and translation (ribosomal proteins) (Supplemental Table S5; Supplemental Fig. S6A,B), supporting the model that IGF2BPs are loaded onto nascent transcripts in the nucleus and shuttle to the cytoplasm (Pan et al. 2007). IGF2BP3 also interacted with signal recognition particle (SRP) components, indicating that IGF2BP3 binds to mRNAs during active translation en route to the ER, consistent with our data. Apart from these factors, our analyses identified an association of IGF2BP3 with the RNA degradation machinery (RNA deadenylation and decapping complex, exosome, XRN1) under both homeostatic and ER stress conditions (Supplemental Table S5; Supplemental Fig. S6A). These data supported the notion that IGF2BP3 accompanies its mRNA targets from synthesis to degradation.

To reduce complexity in our experiments, we aimed to eliminate long-distance RNA-bridged interactors and treated the samples with RNase. RNase treatment reduced the abundance of many interactors, suggesting that they associate with IGF2BP3 through RNA-bridged interactions. Nonetheless, most of its interactors were still detected in the co-IP likely because a large portion of IGF2BP3s is embedded within mRNP complexes that are inaccessible to RNases (Supplemental Fig. S6C). The highly RNase-sensitive interactors were enriched for proteins involved in mRNA splicing, maturation, and export (Supplemental Fig. S6D). RBPs and components of the mRNA degradation machinery showed variable RNase sensitivity. For instance, YBX1, HuR (ELAVL1), G3BP1/2, PABPC1, AGO2, and the LSM complex were highly RNase-sensitive. The RBPs FXR1/2, the RNases IRE1 and XRN1, and the components of the decapping machinery (EDC3, EDC4, DCP1A, and DCP2) were comparatively RNase-resistant, indicating that they are part of more compact assemblies with IGF2BP3 mediated by both protein and RNA interactions (Supplemental Fig. S6C).

**Figure 5.**
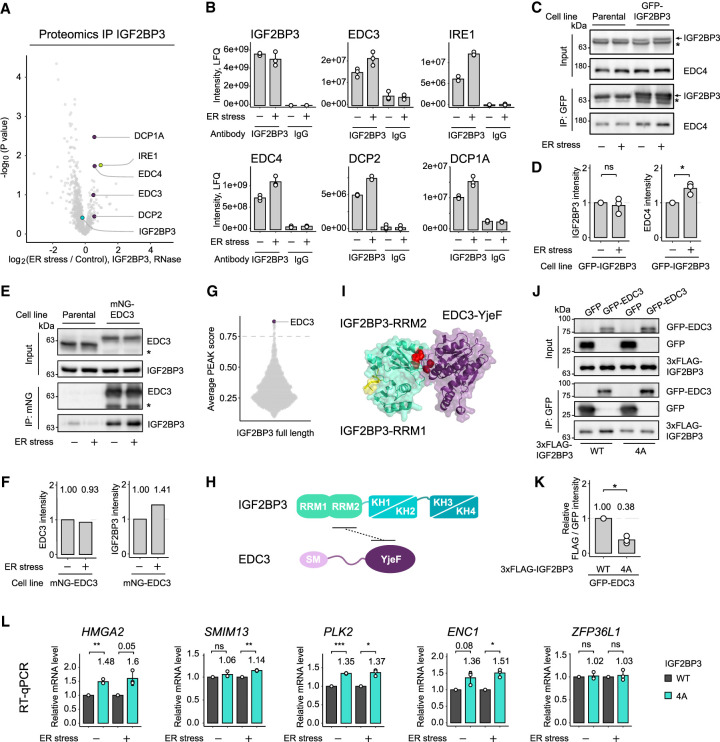
ER stress promotes the association of IGF2BP3 with the mRNA decapping complex. (*A*) Volcano plot comparing interactome of endogenous IGF2BP3 (co-IP–MS analysis) in control and ER stress conditions. Proteins whose interaction with IGF2BP3 is largely resistant to RNase treatment (≤50% reduction) are shown. (*B*) Normalized LFQ values of selected IGF2BP3-interacting proteins showing increased association with IGF2BP3 upon ER stress. Data are the mean ± SD of *n* = 3 biological replicates. (*C*) Western blot showing association of EDC4 with IGF2BP3 upon ER stress after immunoprecipitation of GFP-IGF2BP3 from HEK293T cells. (*) IGF2BP2 isoforms recognized by the polyclonal anti-IGF2BP3 antibody. (*D*) Quantification of *C*. Data are the mean ± SD of *n* = 3 biological replicates. *P*-values were calculated by unpaired two-sided Student's *t*-test. (*E*) Western blot showing association of IGF2BP3 with EDC3 upon ER stress after immunoprecipitation of mNeonGreen-EDC3 from HEK293T cells. (*) Split-mNeonGreen-EDC3 degradation product. (*F*) Quantification of *E*. (*G*) Violin plot of AlphaFold2 Multimer (Jumper et al. 2021; Evans et al. 2022; Mirdita et al. 2022) PEAK scores predicting putative protein–protein interactions between full-length IGF2BP3 and IGF2BP3 interaction partners identified using co-IP-MS. (*H*) Domain architecture of IGF2BP3 and EDC3. (*I*) Predicted structural model (AlphaFold3) (Abramson et al. 2024) of IGF2BP3 RRM1/2 domains (cyan) interacting with EDC3 YjeF domain (violet) through RRM2. RNA interacting with RRM1 is shown in yellow to highlight the RNA interaction interface (Jia et al. 2018). IGF2BP3 residues I88, P90, H91, W94 are shown in red. (*J*) Western blot showing association of IGF2BP3 with EDC3 after immunoprecipitation of GFP-EDC3 from HCT116 IGF2BP3 KO cells expressing 3xFLAG-IGF2BP3 WT or 4A (I88A, P90A, H91A, or W94A) mutant. (*K*) Quantification of *J*. Data are the mean ± SD of *n* = 3 biological replicates. *P*-values were calculated by unpaired two-sided Student's *t*-test. (*L*) RT-qPCR of IGF2BP3 target mRNAs in HCT116 cells expressing IGF2BP3 WT or the IGF2BP3 4A mutant. mRNA levels were normalized to *RPL6* and shown relative to IGF2BP3 WT. Data are the mean ± SD of *n* = 3 biological replicates. *P*-values were calculated by unpaired two-sided Student's *t*-test. For IGF2BP3 co-IP–MS analysis *n* = 3 biological replicates. ER stress was induced with 5 µg/mL TM for 4 h. (*) *P* < 0.05.

While ER stress caused only modest changes in IGF2BP3 binding to the majority of interacting proteins, its association with a subset of proteins, including RNase IRE1, increased (Fig. 5A), supporting our initial findings (Fig. 1A; Supplemental Fig. S1A,B). Notably, during ER stress, IGF2BP3 bound more strongly to all components of the mRNA decapping machinery (EDC3, EDC4, DCP1A, and DCP2) (Fig. 5A,B; Supplemental Fig. S6E), which is in line with the increase of its prodegradatory function during ER stress. We validated this finding by performing co-IP of split-GFP-tagged IGF2BP3 from HEK293T cells. EDC4 was recovered in the IGF2BP3 IP, and its interaction increased upon ER stress, supporting the endogenous co-IP–MS results (Fig. 5C,D). We further corroborated our findings by performing a reciprocal co-IP of another decapping complex component, EDC3, tagged with split-mNeonGreen (mNG), which recovered IGF2BP3 and confirmed its increased association with the decapping complex upon ER stress (Fig. 5E,F). These data suggested that increased interaction of IGF2BP3 with the mRNA decapping machinery might enable more efficient degradation of its target mRNAs during ER stress.

To identify proteins mediating the specific interaction of IGF2BP3 with the mRNA decapping complex, we performed an AlphaFold2 Multimer in silico screen of pairwise protein–protein interactions between IGF2BP3 and confident interactors identified by proteomics analyses (Jumper et al. 2021; Evans et al. 2022; Mirdita et al. 2022). In AlphaFold, a high interface-predicted template modeling score (ipTM score >0.6) indicates high confidence interfaces. Because our data set contained many RBPs with disordered segments, we used the average PEAK score. The average PEAK score quantifies the predicted error in the relative position of two residues and is more sensitive in identifying regions of high confidence for proteins with smaller interaction surfaces and interactions involving disordered regions (Jumper et al. 2021; Evans et al. 2022; Mirdita et al. 2022). The AlphaFold2 Multimer screen identified 14 highly confident IGF2BP3 protein interactors with an average PEAK score >0.75. EDC3 was the second top interactor of IGF2BP3 with an average PEAK score of 0.87 (Fig. 5G). To validate those findings, we performed AlphaFold3 (Abramson et al. 2024) prediction with subdomains of IGF2BP3 and EDC3. IGF2BP3 comprises two N-terminal RNA-recognition motif (RRM) domains and four K homology (KH) domains connected with two disordered linkers (Schneider et al. 2019). EDC3 comprises an N-terminal SM domain connected to the YjeF domain by a long disordered segment (Fig. 5H,I; Tritschler et al. 2007). The AlphaFold3 prediction of the complexes formed between the proteins’ subdomains revealed that the RRM2 domain of IGF2BP3 interacts with the YjeF domain of EDC3 with high confidence (ipTM = 0.72) (Fig. 5H,I; Supplemental Fig. S7A). Importantly, our high-throughput AlphaFold2 Multimer predictions identified the same interaction surface between IGF2BP3 and EDC3 using the full-length proteins, increasing our confidence. In contrast, AlphaFold3 did not predict a confident interaction between other subdomains of both proteins (Supplemental Fig. S7A).

To experimentally validate the binding interface predicted by the structural models, we engineered a quadruple IGF2BP3 mutant (I88A, P90A, H91A, W94A) to impair its binding to EDC3 and called it IGF2BP3-4A mutant (Fig. 5I). We confirmed that IGF2BP3-4A mutant behaves as wild-type IGF2BP3 during purification (Supplemental Fig. S7B). Moreover, we found that purified IGF2BP3-4A displays secondary structure features comparable to those of the wild-type protein (Supplemental Fig. S7C), indicating mutations do not impact IGF2BP3 stability. We next coexpressed GFP-tagged wild-type EDC3 with FLAG-tagged wild-type IGF2BP3 or IGF2BP3-4A in HCT116 IGF2BP3 knockout cells and performed pulldown experiments to assess the interaction. Our data revealed that the IGF2BP3-4A mutant is largely impaired in binding to EDC3 (Fig. 5J,K), indicating that the interaction surface identified by the AlphaFold predictions is essential for IGF2BP3 binding to EDC3. Altogether, we identified a novel interaction between IGF2BP3 and the mRNA decapping complex.

IGF2BP3's KH domains are responsible for RNA recognition, whereas its RRM domains bind RNA with relatively low affinity and specificity (Jia et al. 2018; Schneider et al. 2019). In other RNA-binding proteins, the RRM domains also participate in protein–protein interactions (Maris et al. 2005); we now show that RRM2 mediates IGF2BP3's binding to EDC3 and does not overlap with RNA-binding by the RRM1 domain (Fig. 5I; Jia et al. 2018). Similarly, EDC3 dimerization is mediated by its YjeF domain, and the IGF2BP3 binding does not interfere with its self-association (Ling et al. 2008).

To determine the functional relevance of this interaction, we reintroduced wild-type IGF2BP3 or the IGF2BP3-4A mutant into IGF2BP3 knockout cells via lentiviral transduction. After confirming expression levels comparable to endogenous IGF2BP3 (Supplemental Fig. S7D,E), we evaluated the impact of disrupting the IGF2BP3–EDC3 interaction on target transcripts by RT-qPCR. Expression of the IGF2BP3-4A mutant resulted in higher levels of IGF2BP3 target mRNAs (Fig. 5L). In contrast, it did not affect control mRNAs, which were not destabilized by IGF2BP3 (Supplemental Fig. S7F), supporting our model. Interestingly, *ZFP36L1* levels were unchanged upon disruption of the IGF2BP3–decapping complex interaction, consistent with a proposed miRNA-dependent mechanism for its degradation (Ennajdaoui et al. 2016). To summarize, our findings converge on the model that the increased association of IGF2BP3 with the mRNA decapping complex shifts its function toward promoting target transcript degradation during ER stress.

## Discussion

Among its many essential functions, the ER is the site of folding and maturation of secreted and transmembrane proteins, which together comprise approximately one-third of the proteome. Cells need to rapidly adapt to changes in ER protein-folding demands, as separating protein synthesis and folding into distinct compartments poses a challenge. To overcome this, cells use diverse posttranscriptional and translational mechanisms to adjust the ER protein-folding load. Here, we discovered that IGF2BP3-driven posttranscriptional mechanisms facilitate mRNA degradation and, by indirectly tuning transcription, generate gene regulatory networks that promote UPR signaling while relieving protein-folding load on the ER.

**Figure 6.**
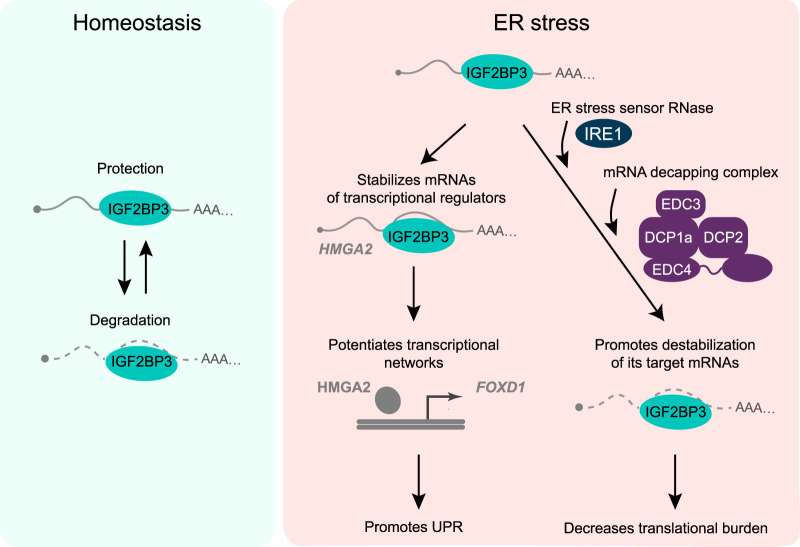
Model of IGF2BP3 function and regulation in ER stress conditions. Depending on the transcript identity, IGF2BP3 binding to its target mRNAs can either promote their degradation or increase stability. Under homeostatic conditions, there is a balance between stabilization and degradation by IGF2BP3. ER stress increases the prodegradation function of IGF2BP3 and degradation of its target transcripts, including the UPR effector mRNAs, through increased interaction of IGF2BP3 with mRNA decapping complexes and, potentially, with the ER stress sensor RNase IRE1. Upon ER stress, IGF2BP3 indirectly elicits a potent UPR by stabilizing mRNA encoding a secondary transcriptional regulator of the UPR, HMGA2. This dual-layered regulation allows IGF2BP3 to specifically upregulate UPR targets while broadly driving degradation of other transcripts to decrease ER folding load until stress is relieved.

Based on our published proteomics data on the interaction between the ER stress sensor RNase IRE1 and IGF2BP3 (Acosta-Alvear et al. 2018), we explored the role of IGF2BP3 in posttranscriptional regulation of mRNA fate during ER stress. Our IR-PAR-CLIP analyses revealed that IGF2BP3 binds to UPR target transcripts during ER stress, including those encoding the major ER chaperones (BiP, calreticulin) and the master UPR transcription factors, XBP1, ATF4, and CHOP. Using complementary approaches to probe for posttranscriptional and transcriptional changes (RNA-seq and SLAMseq) upon IGF2BP3 depletion, we discovered a dual regulatory role, mediated by IGF2BP3-driven posttranscriptional control, that maintains cellular homeostasis during ER stress. The SLAMseq analyses revealed that upon ER stress, IGF2BP3 indirectly promotes transcriptional branches of the UPR, while simultaneously facilitating degradation of its broad mRNA target pool. This IGF2BP3-driven dual regulation helps cells to decrease cellular protein folding burden while enabling them to efficiently express UPR target mRNAs (Fig. 6).

Mechanistically, IGF2BP3 activates the UPR by stabilizing its canonical direct target, *HMGA2*. HMGA2 is a nonhistone chromatin factor that controls gene expression by altering chromatin architecture (Pfannkuche et al. 2009) and supports transcriptional programs linked to embryonic development (Zhou et al. 1995) and oncogenesis (Fedele et al. 1998; Mansoori et al. 2021). Our experiments identify HMGA2 as a key mediator of IGF2BP3-driven transcriptional regulation of UPR genes. Notably, we identified FOXD1, a *forkhead* box transcription factor (Hatini et al. 1996; Quintero-Ronderos and Laissue 2018), as a downstream target of HMGA2 that contributes to UPR activation. The transcriptional branches of the UPR are controlled by a complex network of transcription factors (Jiménez-Beltrán et al. 2026). In line with this, our data show that HMGA2 promotes transcriptional feedback loops and upregulates multiple UPR genes across all three branches. Consistent with our findings, HMGA2 was shown to promote ATF4 transcription (Luo et al. 2023).

In addition to IGF2BP3-mediated regulation of UPR effector RNAs, our data revealed that ER stress leads to a functional switch in IGF2BP3, increasing its destabilizing function. In line with these observations, we discovered that ER stress modulates IGF2BP3 interaction with its canonical targets. IGF2BP3 stabilizes *HMGA2* mRNA by protecting it from miRNA-driven degradation (Jønson et al. 2014). Notably, under mild ER stress, IGF2BP3 protects HMGA2, but under harsher ER stress conditions, IGF2BP3 binding to HMGA2 mRNA decreases, leading to less efficient stabilization and reduced HMGA2 transcript levels. This mechanism may contribute as a negative regulator of the UPR, helping to prevent excessive activation. Beyond the UPR, HMGA2 regulates key programs in embryonic development, stem cell maintenance, and tumorigenesis (Pfannkuche et al. 2009; Ashar et al. 2010; Vignali and Marracci 2020). Therefore, the decrease in HMGA2 levels during ER stress might also affect these processes, suggesting a broader impact of IGF2BP3-mediated regulation under stress.

IGF2BP3 stabilizes its target mRNAs during heat shock (Huang et al. 2018), suggesting that IGF2BP3 can drive opposing outcomes for its target transcripts depending on cellular context. This dynamic and plastic response aligns with the cellular need to adjust transcript levels rapidly during proteotoxic stress. Importantly, IGF2BP3 depletion increased global translational output and protein aggregation under ER stress conditions, highlighting the physiological relevance of IGF2BP3-mediated mRNA destabilization.

Our IP-MS/MS analyses of IGF2BP3 revealed that it strongly associates with all components of the mRNA decapping complex, an interaction that increases by ∼50% during ER stress. Moreover, a high-throughput AlphaFold2 Multimer screen identified a novel interaction surface between EDC3 and IGF2BP3 with high confidence, and an IGF2BP3 mutant engineered to disrupt this interaction was impaired in its binding to EDC3. Supporting our findings, we further show that disrupting IGF2BP3–EDC3 interaction increases the levels of mRNAs destabilized by IGF2BP3. Altogether, we conclude that the increased association of IGF2BP3 with the mRNA decapping complex and RNase IRE1 enhances the degradation of its targets during ER stress. Future work will provide insights into the mechanisms that mediate the preferred association of the mRNA decapping complex with IGF2BP3 during ER stress.

IGF2BP3 is an essential regulator of early development, and its overexpression in various tumors correlates with poor prognosis and cancer aggressiveness (Lederer et al. 2014; Mizutani et al. 2016; Xu et al. 2019). Similarly, physiological UPR contributes to development and differentiation, while cancer cells hijack this process to promote their growth (Salvagno et al. 2022). We hypothesize that the dual-layered regulation we have uncovered allows cells to shift between opposing IGF2BP3 regulatory effects on UPR targets, implying a cell state-specific regulation. While this regulatory plasticity may contribute to exquisite regulation during development, the balance may be tipped in cancer and impact UPR-driven cell fate decisions, presenting a novel avenue for therapeutic targeting. To sum up, we discovered a novel IGF2BP3-mediated dual regulatory mechanism that ensures an adaptive response to ER stress.

## Materials and methods

### Mammalian cell culture

HCT116 WT cells were a kind gift from the Manuela Baccarini Laboratory (Max Perutz Labs). HCT116 cells conditionally expressing doxycycline-inducible (tetON) OsTIR1 were obtained from the Masato Kanemaki laboratory (Natsume et al. 2016). RKO cells expressing doxycycline-inducible Cas9 were a kind gift from Johannes Zuber (IMP) (de Almeida et al. 2021). HEK293T cells expressing splitGFP-IGF2BP3 were a kind gift of Manuel Leonetti established using published protocols (Leonetti et al. 2016). HEK293T cells expressing split mNeonGreen-EDC3 were a kind gift of Manuel Leonetti (Chen-Zuckerberg Biohub [Cho et al. 2022]).

HCT116 cells were cultured in McCoy's 5A (modified) medium (Sigma M9309) supplemented with 10% fetal bovine serum (Gibco 10437028), 2 mM l-glutamine (Sigma G7513), 1% Pen/Strep (Sigma P0781). HEK293T cells were cultured in high-glucose DMEM media (Sigma D5796) supplemented as above. RKO cells were cultured in RPMI-1640 media (Sigma R8758) supplemented with 10% fetal bovine serum (Gibco 10437028), 2 mM l-glutamine (Sigma G7513), 1% Pen/Strep (Sigma P0781), 1× nonessential amino acids (Thermo Scientific 11140050), and 2 mM sodium pyruvate (Gibco 11360070). All cell lines were cultured in a humidified incubator at 37°C and 5% CO_2_ and regularly tested for mycoplasma infection with the EZ-PCR mycoplasma detection kit (Biological Industries).

### Western blotting

Eighty percent of confluent cells were lysed with RIPA buffer (150 mM NaCl, 1% NP-40, 0.5% sodium deoxycholate, 0.1% SDS, and 25 mM TRIS at pH 7.4) with 1× EDTA-free protease inhibitor cocktail (Roche). Lysates were clarified using a tabletop centrifuge at maximum speed (20,000*g*) for 20 min at 4°C. Western blot samples were denatured at 95°C for 5 min in 1× SDS sample buffer (50 mM Tris-HCl at pH 6.8, 2% SDS, 0.1% Bromophenol blue, 10% glycerol, 20 mM DTT). Following denaturing, the samples were loaded onto a 10%, 12%, or 15% sodium dodecyl sulfate (SDS) gel. Proteins were transferred onto a 0.2 µm nitrocellulose membrane (Amersham) using BioRad Trans-Blot Turbo transfer system or with wet transfer in transfer buffer (25 mM TRIS, 190 mM glycine, 20% ethanol) for 110 min at 120 V. Membranes were stained with Ponceau S and blocked in 5% milk for 1 h. The primary antibody (Supplemental Table S6) was diluted in 2.5% milk and incubated overnight at 4°C. The membrane was washed five times with TBST (20 mM TRIS, 150 mM NaCl, 0.1% Tween 20), and the membranes were incubated for 1 h with the secondary antibody (Supplemental Table S6) diluted in 2.5% milk. After the incubation the membranes were washed five times with TBST. Membranes were developed with enhanced chemiluminescent (ECL) horseradish peroxidase substrate (Westar ETA C Ultra 2.0, Cyanagen), imaged using BioRad ChemiDoc, and analyzed using ImageLab software (6.1.0). Near-infrared Western blotting was performed as described above with the following modifications. Membranes were blocked in Intercept (TBS) Blocking Buffer (LICORbio). Primary antibodies were diluted in Intercept (TBS) blocking buffer (LICORbio) supplemented with 0.15% Tween 20, and secondary antibodies (IRDye 800CW goat antirabbit, LICORbio) in Intercept T20 (TBS) antibody diluent (LICORbio). Before imaging, membranes were washed an additional three times in TBS for 5 min each and imaged on Li-Cor Odyssey CLx fluorescence imager.

### IR-PAR-CLIP of IGF2BP3—sequencing library preparation and data processing

The detailed protocol for the infrared (IR) PAR-CLIP of IGF2BP3 is described by Anisimova and Karagöz (2023). One-hundred-twenty-five million (five 15 cm dishes) HCT116 inducible tetON OsTIR1 cells were used per condition. 4sU (100 µM) (Sigma) was added to the cell culture media 15 h prior to collection. ER stress was induced with 5 µg/mL TM for 4 h prior to collection. DMSO at a 1:1000 dilution was used as a control. IGF2BP3 was immunoprecipitated with anti-IGF2BP3 Proteintech antibody (14642-1-AP, lot 00088732). anti-IgG Proteintech antibody (30000-0-AP) was used as a control. RNase I (Ambion) was used for the in-lysate RNase digestion at 0.1 U/µL and for the on-bead digestion in 1 mL of the lysis buffer at 0.025 U/µL. IR-PAR-CLIP libraries were sequenced on a NovaSeq 6000 S1 on SR100 (Illumina) at the Vienna BioCenter Next-Generation Sequencing Facility with 55 million reads per sample on average. UMIs were extracted and adapter sequences were trimmed using UMI-tools v1.1.1 (Smith et al. 2017). The reads were size- and quality-trimmed using Trimmomatic v0.30 (Bolger et al. 2014) to have a length between 18 and 45 nt. The reads were then mapped to human genome hg38 using GENCODE annotation (release 36) with bowtie v0.12.7 (Langmead et al. 2009), allowing up to three mismatches and deduplicated using UMI-tools v1.1.1. Gene counts were obtained using the FeatureCounts function of the Subread package v2.0.1 (Liao et al. 2014). Gene counts for protein coding genes were RLE normalized to calculate the counts per million values (CPM), filtered to only include genes with CPM higher than 5 in at least one-third of the libraries, and the differential expression analysis was performed with edgeR glmQLFTest (generalized linear model quasilikelihood *F*-test) (Robinson et al. 2010). GO term analysis was performed with Enrichr webserver (Chen et al. 2013; Kuleshov et al. 2016). Redundancy in GO terms was reduced using GOSemSim (Yu et al. 2010) and rrvgo (Sayols 2023). Full GO term enrichment lists are shown in the Supplemental Material.

### IR-PAR-CLIP of IGF2BP3—matching transcriptome library preparation and data processing

An aliquot of the lysate was taken before the in-lysate RNase digest for total RNA isolation and sequencing. Total RNA was isolated using peqGOLD TriFast (Peqlab, VWR) and transcriptome libraries were prepared with QuantSeq 3′ mRNA-seq library preparation kit (Lexogen). Libraries were sequenced on a NextSeq2000 P2 at SR100 mode (Illumina) at the Vienna BioCenter Next-Generation Sequencing Facility with 30 million reads per sample on average. The quality-, adapter-, and polyA-trimmed reads were aligned to human genome hg38 using GENCODE annotation (release 36) with STAR v2.7.5c. As the reads originate from cross-linked PAR-CLIP samples and have T to C transitions, mismatches were allowed. Gene counts for protein coding genes were RLE normalized to calculate the counts per million values (CPM), filtered to only include genes with a CPM higher than 1 in at least half of the libraries. Differential expression analysis was performed with edgeR glmQLFTest (Robinson and Oshlack 2010).

### IR-PAR-CLIP of IGF2BP3—computational analysis

To estimate relative binding of IGF2BP3, IR-PAR-CLIP CPMs were divided to the matching total transcriptome QuantSeq CPMs. A pseudocount of 1 CPM was added to all samples and only genes with mean CPM for QuantSeq samples >5 CPM were taken for the analysis. To identify IGF2BP3 target genes, IGF2BP3-binding clusters were called with PARalyzer v1.5 (ini file is available in Supplemental Table S6; Corcoran et al. 2011). Genes that had at least one cluster containing more than 25 CPM and more than 50% of T to C conversions per read in two replicates (nos. 2 and 3) per condition were selected as IGF2BP3 targets. Samples from replicate 1 were excluded from target identification due to lower final read number and therefore lower number of identified clusters and targets. The target lists were filtered to only include targets with PAR-CLIP CPMs higher than total transcriptome QuantSeq CPMs. PAR-CLIP genomic tracks were visualized using svist4get v1.2.20 (Egorov et al. 2019). The enrichment of 5 nt motifs in IGF2BP3 PAR-CLIP reads was analyzed with HOMER v4.11 (Heinz et al. 2010), findMotifs.pl command using shuffled background. m6A and m7G motif enrichment analysis was done for IR-PAR-CLIP reads (replicates were pooled per condition) using MEME suite SEA (simple enrichment analysis) (Bailey and Grant 2021). Motif enrichment was calculated either over shuffled sequences or pooled IR-PAR-CLIP reads for particular condition (indicated in the figure legends). Positions of methylation sites for *HSPA5* were mapped based on miCLIP-seq data from m6A Atlas 2.0 (data sets GSE152633 [Cho et al. 2021], GSE161304 [Cotter et al. 2021], GSE137675 [Jia et al. 2019], GSE98623 [Vu et al. 2017] from m6A and m7G miCLIP-seq [Malbec et al. 2019]; Liang et al. 2024). Sequencing data processing was done using the HPC of the Center for Integrative Bioinformatics Vienna (CIBIV) and Life Science Compute Cluster (LiSC) of the University of Vienna, Austria.

### Establishment of IGF2BP3 knockout cell lines and siRNA knockdown

For IGF2BP3 knockout cell line generation, gRNA sequences (clones E4 and A3 [5′-TGGCACCGACTGATAGAGCT-3′] and clone D12 [5′-ACGCGTAGCCAGTCTTCACC-3′]) were cloned into the pSpCas9 (BB)-2A-GFP (PX458) (plasmid 48138, Addgene) (Ran et al. 2013). HCT116 tetON OsTIR1 cells were transiently transfected using jetOPTIMUS reagent (Tamar 101000051), and GFP-positive single-cell clones were FACS sorted at BD FACSAria IIIu at Max Perutz Labs BioOptics FACS Facility. HCT116 cells do not express IGF2BP1 (Mongroo et al. 2011). For siRNA knockdown, HCT116 cells (WT or tetON OsTIR1) or RKO iCas9 were transfected with IGF2BP3 (Dharmacon L-003976-00-0005) SMARTpool siRNAs using DharmaFECT 2 (Dharmacon T-2002-01) at 75 nM for 48 h. For depletion of HMGA2 and FOXD1, cells were transfected with SMARTpool siRNAs (Dharmacon) targeting HMGA2 (L-013495-00-0005) and FOXD1 (J-011862-00-0005) at 50 nM for 48 h. ON-TARGETplus nontargeting siRNA pool (Dharmacon D-001810-10-05) was used as a control.

### Establishment of doxycycline-inducible IGF2BP3 knockout RKO cell lines

RKO-Dox-Cas9 (iCas9) cell lines for doxycycline-inducible knockout IGF2BP3 were established using lentiviral transduction with lentiviral particles (produced as described by Hornegger et al. 2024) containing dual-sgRNA_hU6-mU6 vectors described by de Almeida et al. (2021) expressing two sgRNAs against IGF2BP3 (5′-TGGCACCGACTGATAGAGCT-3′ and 5′-GAAGATACTTTCTAGGTCCG-3′) or against noncoding locus AAVS1 (5′-CGCTGTGCCCCGATGCACAC-3′ and 5′-GGCGCGTCGCTCGCTCGCTC-3′) from human and mouse U6 promoters and eBFP2 from a PGK promoter. The eBFP2-positive cells were FACS sorted at the BD FACSMelody Cell Sorter at the Max Perutz Labs BioOptics FACS Facility.

### Establishment of HCT116 cell lines expressing IGF2BP3 4A mutant

For establishment of HCT116 cell lines expressing IGF2BP3 WT and 4A mutant at endogenous levels, HCT116 tetON OsTIR1 IGF2BP3 KO (clone A3) cells were transduced with lentiviral particles (produced and transduced as described in Hornegger et al. 2024) containing the plenti-CAG-IRES-GFP vector (Addgene 69047) (Lu et al. 2014a) with full-length IGF2BP3 WT and 4A mutant (I88A, P90A, H91A, W94A). IGF2BP3 sequences were cloned into the plenti-CAG-IRES-GFP vector using EcoRI and XhoI restriction sites. To achieve expression levels comparable to endogenous IGF2BP3, GFP-positive cell populations were gated for high, middle, and low GFP expression levels and sorted in two consecutive rounds on the Cytek Aurora CS Cell Sorter at the Max Perutz Labs BioOptics FACS Facility. The expression levels of IGF2BP3 WT and 4A mutant were compared to those in the HCT116 tetON OsTIR1 cell line using near-infrared Western blotting, and populations with endogenous IGF2BP3 WT and 4A mutant levels were selected.

### Total transcriptome sequencing (RNA-seq) of IGF2BP3 knockout HCT116 cells

HCT116 tetON OsTIR1 IGF2BP3 knockout clones E4, A3, and D12 (in duplicates) and parental cells (four replicates) were treated with 5 µg/mL TM for 4 h (1:1000 DMSO was used as a control). Total RNA was isolated using peqGold TriFast (Peqlab, VWR), treated with RNase-free DNase I (NEB), and repurified with peqGold TriFast (Peqlab, VWR). RNA precipitation was done using the isopropanol method. Total RNA sequencing libraries were prepared with NEBNext poly(A) mRNA magnetic isolation module (NEB E7490L) and NEBNext Ultra II directional RNA library preparation kit (NEB E7765L) by the Vienna BioCenter Next-Generation Sequencing Facility sequenced on a NovaSeq S4 on PE100 (Illumina) with 55 million reads per sample on average. The quality- and adapter-trimmed reads were aligned to human genome hg38 using Gencode annotation (release 36) with STAR v2.7.5c allowing up to two mismatches per read. Gene counts for protein coding genes were RLE normalized to calculate the counts per million values (CPM), filtered to only include genes with CPM >5 in at least half of the libraries, and differential expression analysis was performed with edgeR glmQLFTest (Robinson and Oshlack 2010).

### RNA-seq of HCT116 cells upon siRNA-mediated depletion of IGF2BP3

HCT116 WT cells were depleted of IGF2BP3 using siRNA pools as described above in “Establishment of IGF2BP3 Knockout Cell Lines and siRNA Knockdown.” ER stress was induced with 5 µg/mL TM for 4 h prior to collection. One ∼80% confluent well of a 6 well plate per condition (three biological replicates per condition) was washed with ice-cold PBS (Sigma) supplemented with 100 mg/mL cycloheximide, lysed on a plate on ice with 350 µL of ice-cold lysis buffer (20 mM HEPES at pH 7.3, 150 mM KCl, 5 mM MgCl_2_, 1% Triton X-100, 100 mg/mL cycloheximide, 1 mM DTT, 1× EDTA-free protease inhibitor cocktail), scraped, and transferred to a 1.5 mL RNase-free tube. Cell lysates were passed three times through the 27G needle, incubated on ice for 10 min with intermittent vortexing, and clarified on a tabletop centrifuge at maximum speed (20,000*g*) for 20 min at 4°C. Total RNA was extracted from 15 µL of the lysate using KingFisher Flex purification system (Thermo) with the high-performance RNA isolation kit (Molecular Tools Shop, Vienna BioCenter). RNA-seq libraries were prepared with NEBNext poly(A) mRNA magnetic isolation module (NEB E7490L) and NEBNext Ultra II directional RNA library preparation kit (NEB E7765L) and sequenced on a NovaSeq SP on SR100 (Illumina) with 30 million reads per sample on average. The quality- and adapter-trimmed reads were aligned to human genome hg38 using GENCODE annotation (release 36) with STAR v2.7.5c allowing up to two mismatches per read. Gene counts for protein coding genes were filtered to include genes with coding sequence length-normalized CPM values >5 in at least one-third of the libraries. Differential expression analysis was performed with edgeR glmQLFTest (Robinson and Oshlack 2010).

### Comparison of the RKO transcriptome upon doxycycline-inducible Cas9- and siRNA-mediated depletion of IGF2BP3

IGF2BP3 depletion was induced in RKO iCas9 cells with 250 ng/mL doxycycline for 72 h. siRNA-mediated depletion was performed in RKO iCas9 as described above in “Establishment of IGF2BP3 Knockout Cell Lines and siRNA Knockdown.” RKO transcriptome was obtained using SLAMseq protocol (Herzog et al. 2017). 4-Thiouridine (s^4^U) was added to cell culture media at 250 µM 2 h prior to collection. Total RNA was extracted using the KingFisher Flex Purification System (Thermo) with the high-performance RNA isolation kit (Molecular Tools Shop, Vienna BioCenter). During the isolation RNA was treated with RNase-free DNase I (NEB). Samples were prepared according to the standard SLAMseq protocol described in Herzog et al. (2017). Briefly, total RNA was alkylated with 10 mM iodoacetamide in alkylation buffer (50 mM sodium phosphate buffer at pH 8.0, 50% DMSO) for 15 min at 50°C and purified using the KingFisher Flex purification system. Sequencing libraries were prepared with NEBNext poly(A) mRNA magnetic isolation module (NEB E7490L) and NEBNext Ultra II directional RNA library preparation kit (NEB E7765L) and sequenced on a NovaSeq SP on SR100 (Illumina) at the Vienna BioCenter Next-Generation Sequencing Facility with 30 million reads per sample on average. The quality- and adapter-trimmed reads were aligned to human genome hg38 using Gencode annotation (release 36) with STAR v2.7.5c allowing up to five mismatches per read. Total gene counts for protein coding genes were analyzed and genes were filtered to include those with coding sequence length-normalized CPM >5 in at least half of the libraries. Differential expression analysis was performed with edgeR glmQLFTest (Robinson and Oshlack 2010). The data were processed using the HPC of the Center for Integrative Bioinformatics Vienna (CIBIV).

### Identification of siRNA pool off-targets from siRNA knockdown experiments

To exclude possible siRNA off-target genes we compared changes in gene expression upon doxycycline-inducible knockout and siRNA-mediated depletion of IGF2BP3 in RKO iCas9 cells. Genes were considered off-targets if changes in their levels were more than 20% upon siRNA-mediated depletion, but <10% upon doxycycline-inducible knockout. Significance was not considered for this analysis due to overall small amplitude of changes. The identified off-targets are listed in Supplemental Table S2. These genes were excluded from analyses of experiments where IGF2BP3 was depleted with siRNA pools.

### SLAMseq of HCT116 upon siRNA-mediated depletion of IGF2BP3

HCT116 WT cells were depleted of IGF2BP3 using siRNA pools as described in “Establishment of IGF2BP3 Knockout Cell Lines and siRNA Knockdown.” ER stress was induced using a 5 h-long treatment with 5 µg/mL TM (1:1000 DMSO was used as a control). 4-Thiouridine (s^4^U) was added to cell culture media at 250 µM 2 h prior to collection. Total RNA was extracted using the KingFisher Flex purification system (Thermo) with the high-performance RNA isolation kit (Molecular Tools Shop, Vienna BioCenter). During the isolation RNA was treated with RNase-free DNase I (NEB). SLAMseq samples were prepared according to the standard SLAMseq protocol described in Herzog et al. (2017). Briefly, total RNA was alkylated with 10 mM iodoacetamide in alkylation buffer (50 mM sodium phosphate buffer at pH 8.0, 50% DMSO) at 50°C for 15 min and purified using the KingFisher Flex purification system. Sequencing libraries were prepared with NEBNext poly(A) mRNA magnetic isolation module (NEB E7490L) and NEBNext Ultra II directional RNA library preparation kit (NEB E7765L) and sequenced on a NovaSeq SP on SR100 (Illumina) at the Vienna BioCenter Next-Generation Sequencing Facility with 30 million reads per sample on average. The SLAMseq fastq files were trimmed and aligned to human genome hg38 using the conversion-aware nf-core/slamseq analysis pipeline v1.0.0 (Neumann et al. 2019; Ewels et al. 2020) with default parameters. T-C conversion rates were calculated using the *slamdunk* pipeline (Neumann et al. 2019). The T-C conversion rate values are ratios of T-C conversions to the total T coverage at each T-position within annotated 3′ UTR. Position-wise conversion rates were averaged across all T positions per gene to obtain a gene-level T-C conversion rate. T-C conversion rates reflect the fraction of newly synthesized RNA and inversely correlate with mRNA stability estimates. Genes were filtered to only include those with both total and T-C CPM >5 for at least half of the samples. The differential expression analysis was performed with edgeR glmQLFTest (Robinson and Oshlack 2010). Sample siControl ER stress replicate 3 was a clear outlier on a PCA plot and was excluded from analysis. The data were processed using the Life Science Compute Cluster (LiSC) of the University of Vienna.

### RNA isolation and RT-qPCR

Total RNA was isolated using the KingFisher Flex purification system (Thermo) with the high-performance RNA isolation kit (Molecular Tools Shop, Vienna BioCenter). During the isolation RNA was treated with DNase I (NEB M0303S). cDNA was prepared with LunaScript RT SuperMix (NEB) and amplified in a qPCR reaction with 2× Hot Start qPCR master mix (Molecular Tools Shop, Vienna BioCenter) using Bio-Rad CFX 384 Touch machine. The qPCR primers are listed in Supplemental Table S6. mRNA levels were calculated relative to *RPL6* levels. mRNA decay rate constants and half-lives were calculated from ordinary least-squares linear regression of ln-transformed mRNA levels over time following actinomycin D treatment, as described previously (Chen et al. 2008).

### Puromycin incorporation assay

HCT116 WT cells were depleted of IGF2BP3 using siRNA pools as described above in “Establishment of IGF2BP3 Knockout Cell Lines and siRNA Knockdown” or transfected with control nontargeting siRNA. One well from a 24 well plate was used per condition. ER stress was induced by a 4 h treatment with 5 µg/mL TM (1:1000 DMSO was used as a control). Cells were treated with 20 µg/mL puromycin 30 min before collection. Cells were collected in 100 µL of RIPA buffer (150 mM NaCl, 1% NP-40, 0.5% sodium deoxycholate, 0.1% SDS, 25 mM TRIS at pH 7.4) with 1× EDTA-free protease inhibitor cocktail (Roche). Lysates were clarified using a tabletop centrifuge at maximum speed (20,000*g*) for 20 min at 4°C. Samples for Western blot were prepared as described above in “Western Blotting.”

### Aggresome detection assay

Aggresome detection was performed using Enzo PROTEOSTAT Aggresome Detection Kit (ENZ-51035-0025). HCT116 WT cells were depleted of IGF2BP3 using siRNA pools as described in “Establishment of IGF2BP3 Knockout Cell Lines and siRNA Knockdown,” or transfected with control nontargeting siRNA. One well from a 6 well plate was used per condition. ER stress was induced by a 4 h treatment with 5 µg/mL TM (1:1000 DMSO was used as a control). Cells were washed in cold PBS and collected by trypsinization. Cells were pelleted by centrifugation at 400*g* for 5 min, and the pellet was washed twice with PBS. At the last wash, cells were resuspended in ∼200 µL of remaining PBS and fixed by adding the cell suspension dropwise into 1 mL of 4% formaldehyde solution in PBS and incubated for 30 min at room temperature. Fixed cells were collected by centrifugation at 800*g* for 15 min and washed once with 1 mL of PBS. After centrifugation at 800*g* for 15 min, supernatant was discarded, cells were permeabilized by adding the cell suspension into 1 mL of cell permeabilization buffer (0.5% Triton X-100, 3 mM EDTA at pH 8, in 1× commercial assay buffer from Enzo Proteostat aggresome detection kit) and incubated for 30 min on ice. Cells were collected by centrifugation at 800*g* for 15 min at 4°C, supernatant was discarded, and cells were washed with 1 mL of PBS. The resulting cell suspension was filtered through the strainer cap of a Fluorescence-activated cell sorting (FACS)-compatible BD tube and the suspension was centrifuged at 800*g* for 15 min at 4°C. After removing supernatant, remaining cell pellet was stained by resuspending it in 500 µL of freshly diluted 1:200 Proteostat aggresome detection reagent in 1× assay buffer from the kit. After 30 min incubation at room temperature protected from light, the samples were analyzed by FACS on Bio-Rad ZE5 cell analyzer in 488 nm excitation and 583/52 nm emission filter.

### Total proteome of IGF2BP3 KO HCT116 cells—sample preparation

Parental HCT116 tetON OsTIR1 cells and IGF2BP3 full knockout (clone A3) cells were grown to ∼70% confluency and treated with 100 ng/mL TM for 8 h (1:50,000 DMSO was used as a control). Cells were washed twice with 10 mL of ice-cold PBS, collected by scraping and pelleting at 500*g* for 5 min at 4°C, flash-frozen in liquid nitrogen, and stored at −80°C. Four replicates per condition were collected. The cell pellets were lysed in heated 2% sodium deoxycholate and 0.1 m Tris/HCl (pH 8.8) for 10 min at 95°C and then sonicated in a Bioruptor for five cycles 30/30 sec with level H in a cold room. After the supernatant cooled down, 1 µL of benzonase was added and pellets were incubated for 30 min on ice followed by another sonication in a Bioruptor for five cycles 30/30 sec with level H in a cold room. The supernatant was kept on ice for another 10 min before centrifugation at 15,000*g* for 10 min at 10°C to pellet cell debris. The supernatant was transferred to a new tube, and the protein concentration was measured with a microBCA assay (Thermo Fisher). About 50 µg of protein in 25 µL was reduced with 1 µL of 250 mM dithiothreitol (DTT) for 30 min at 50°C before adding 1 µL of 500 mM iodoacetamide and incubating for 30 min at room temperature in the dark. Remaining iodoacetamide was quenched with 0.5 µL of 250 mM DTT for 10 min. The solution was diluted to 1% sodium deoxycholate using 25 µL 50 mM ammonium bicarbonate. Proteins were digested with 0.5 µg of LysC (mass spectrometry grade; FujiFilm Wako chemicals) in 0.5 µL of 50 mM ammonium bicarbonate for 3 h at room temperature and then digested further with 1 µg of trypsin (Trypsin Gold, Promega) in 1 µL of 50 mM ammonium bicarbonate overnight at 37°C. The digest was stopped by the addition of 10% trifluoroacetic acid (TFA) to a final concentration of 2%. Then the samples were centrifuged at 14,000 rpm for 10 min, and the supernatant was transferred to a new tube and desalted using a 96 well Oasis MCX µElution plate (Waters, 30 µm particle size, 186001830BA) following the manufacturer's protocol.

### Total proteome of IGF2BP3 KO HCT116 cells—liquid chromatography-mass spectrometry

LC-MS analysis was performed on a Vanquish Neo UHPLC system (Thermo Scientific) coupled to an Orbitrap Astral mass spectrometer (Thermo Scientific). The system was equipped with a Nanospray Flex ion source (Thermo Scientific), and a Column Heater (IonOpticks) connected to a Heater Controller (IonOpticks). Peptides were loaded onto a trap column (PepMap Neo C18 5 mm × 300 µm, 5 µm particle size, Thermo Scientific) using 0.1% TFA as mobile phase, and separated on an analytical column (Aurora Ultimate XT C18, 25 cm × 75 µm, 1.7 µm particle size, IonOpticks), applying a linear gradient starting with a mobile phase of 98% solvent A (0.1% FA) and 2% solvent B (80% acetonitrile, 0.08% FA), increasing to 35% solvent B over 60 min at a flow rate of 300 nL/min. The analytical column was heated to 50°C. The mass spectrometer was operated in data-independent acquisition (DIA) mode. Survey scans were acquired from 380 to 980 m/z, normalized AGC target of 300%, resolution of 240,000 in the Orbitrap with a maximum 5 msec injection time. In total, 149 MS2 spectra were acquired across a mass range of 380.4–980.7 m/z using an isolation window of 4 m/z with 0 m/z overlap between windows. Window placement optimization is on. Selected ions were analyzed using a maximum injection time of 5 msec, normalized AGC target of 500% after HCD fragmentation with normalized collision energy of 25% in the Astral detector. MS2 scan range is 150–2000. The loop control is time with a 0.6 sec cycle.

### Total proteome of IGF2BP3 KO HCT116 cells—mass spectrometry data analysis

MS raw data were processed with Spectronaut (20.4, Biognosys). The library-free DirectDIA+ workflow was employed for analysis of the raw files, the *Homo sapiens* one protein per gene reference proteome from UniProt (Proteome ID: UP000005640, release 2025_01), concatenated with a database of 379 common laboratory contaminants (release 2025_01, https://www.github.com/maxperutzlabs-ms/perutz-ms-contaminants), and an entry for P17861-2. The cleavage specificity was set to full trypsin specificity (Trypsin/P), with two missed cleavages allowed. Carbamidomethyl was used as fixed cysteine modification; methionine oxidation and protein N-terminal acetylation were specified as variable modifications. Cross-run normalization was disabled, and all other settings were used at their default values.

Computational analysis was performed using Python and the in-house developed Python library MsReport (0.0.32) (Hollenstein et al. 2023). Only noncontaminant proteins identified with a minimum of two peptides and being quantified in at least two replicates of one condition were considered for further analysis. LFQ protein intensities were log_2_-transformed and normalized across samples using the ModeNormalizer from MsReport. The ModeNormalizer method involves calculating log_2_ protein ratios for all pairs of samples and determining normalization factors based on the modes of all ratio distributions. Missing values were imputed by drawing random values from a normal distribution. The σ and μ of this distribution were calculated per sample from the standard deviation and median of the observed log_2_ protein intensities (µ = median sample LFQ intensity − 1.8 standard deviations of the sample LFQ intensities and σ = 0.3 × standard deviation of the sample LFQ intensities). iBAQ intensities were calculated by dividing protein intensities by the number of theoretically observable tryptic peptides between 6 and 30 amino acids. To estimate the relative protein abundances within each sample, the iBAQ intensities were normalized by dividing them by the total sum of iBAQ intensities for all proteins in the sample. Statistical analysis was performed using the linear models for microarray analysis (limma) v.3.54.2 (Ritchie et al. 2015) package in R. Moderated *t*-statistics were calculated using the limma-trend method, and multiple testing correction was applied using the Benjamini–Hochberg method. The Python library XlsxReport (0.1.1) was used to create formatted Excel files summarizing the results of the proteomics experiments (Supplemental Table S3).

### Coimmunoprecipitation of endogenous IGF2BP3 followed by mass spectrometry—sample preparation

One 15 cm dish of 80% confluent HCT116 WT cells was used per condition. To induce ER stress, cells were treated with 5 µg/mL TM for 4 h, and DMSO at 1:1000 dilution was used as a control. Cells were washed with ice-cold PBS and collected by scraping and pelleting at 500*g* for 5 min at 4°C. Cells were lysed in 250 µL of lysis buffer (25 mM HEPES at pH 7.3, 150 mM NaCl, 0.5% NP-40, 0.5 mM EDTA, 10% Glycerol, 1× EDTA-free protease inhibitor cocktail, 1× PhosSTOP) per condition by incubation on ice 15 min with intermittent vortexing. The lysate was clarified by centrifugation at 20,000*g* for 20 min at 4°C. Ten micrograms of Proteintech anti-IGF2BP3 antibody (14642-1-AP, lot 00088732) or Proteintech IgG control (30000-0-AP) was coupled to the 40 µL of protein G Dynabeads (Invitrogen) in 1 mL of lysis buffer for 20 min, rotating at room temperature, washed three times with 1 mL of the lysis buffer, resuspended in the original bead volume (40 µL) and added to 200 µL of the clarified lysate. Beads were washed five times with 1 mL of wash buffer (25 mM HEPES at pH 7.3, 150 mM NaCl, 0.5 mM EDTA, 10% glycerol). Each wash was incubated on ice for 3 min. For RNase digestion 0.1 U/µL RNase I (Ambion) and 1 U/µL RNase T1 (EN0541) were added to the second wash. Samples were incubated at room temperature for 10 min on a rotator. The control (undigested) samples were kept on ice. The proteins were eluted from the beads with 20 µL of 100 mM glycine (pH 2.0) three times and the pooled supernatant was adjusted to alkaline using about 20 µL of 1 m Tris (pH 8.0). Disulfide bonds were reduced with 3.2 µL of 250 mM dithiothreitol (DTT) for 30 min at room temperature before adding 3.2 µL of 500 mM iodoacetamide and incubating for 30 min at room temperature in the dark. Remaining iodoacetamide was quenched with 1.6 µL of 250 mM DTT for 10 min. Proteins were digested with 300 ng trypsin (Trypsin Gold, Promega) in 3 µL of 50 mM ammonium bicarbonate overnight at 37°C. The digest was stopped by the addition of 10% trifluoroacetic acid (TFA) to a final concentration of 0.5%. The peptides were desalted using C18 Stagetips (Rappsilber et al. 2007).

### Coimmunoprecipitation of endogenous IGF2BP3 followed by mass spectrometry—liquid chromatography-mass spectrometry analysis

Peptides were separated on a Vanquish Neo nano-flow chromatography system (Thermo Fisher), using a trap-elute method for sample loading (Acclaim PepMap C18, 2 cm × 0.1 mm, 5 µm, Thermo Fisher), and a C18 analytical column (Acclaim PepMap C18, 50 cm × 0.075 mm, 2 µm, Thermo Fisher), applying a segmented linear gradient from 2% to 35% and finally 80% solvent B (80% acetonitrile, 0.1% formic acid; solvent A 0.1% formic acid) at a flow rate of 230 nL/min over 120 min. Eluting peptides were analyzed on an Exploris 480 Orbitrap mass spectrometer (Thermo Fisher Scientific) coupled to the column with a FAIMS Pro ion source (Thermo Fisher Scientific) using coated emitter tips (PepSep, MSWil) with the following settings: The mass spectrometer was operated in DDA mode with two FAIMS compensation voltages (CV) set to −45 or −60 and 1.5 sec cycle time per CV. The survey scans were obtained in a mass range of 350–1500 *m/z*, at a resolution of 60,000 at 200 *m/z*, and a normalized AGC target at 100%. The most intense ions were selected with an isolation width of 1.4 *m/z*, fragmented in the HCD cell at 30% collision energy, and the spectra were recorded for a maximum of 50 msec at a normalized AGC target of 100% and a resolution of 15,000. Peptides with a charge of +2 to +6 were included for fragmentation, the peptide match feature was set to preferred, the exclude isotope feature was enabled, and selected precursors were dynamically excluded from repeated sampling for 45 sec.

### Coimmunoprecipitation of endogenous IGF2BP3 followed by mass spectrometry—proteomics data analysis

The RAW MS data were analyzed with FragPipe (20.0), using MSFragger (4.1) (Kong et al. 2017). IonQuant (1.10.27) (Yu et al. 2021) and Philosopher (5.0.0) (da Veiga Leprevost et al. 2020). The default FragPipe workflow for label free quantification (LFQ-MBR) was used, except “normalize intensity across runs” was turned off. Cleavage specificity was set to Trypsin/P, with two missed cleavages allowed. The protein FDR was set to 1%. A mass of 57.02146 (carbamidomethyl) was used as fixed cysteine modification; methionine oxidation and protein N-terminal acetylation were specified as variable modifications. MS2 spectra were searched against the human one protein per gene reference proteome from UniProt (Proteome ID: UP000005640, release 2024_01), concatenated with a database of 382 common laboratory contaminants (release 2023.03, https://www.github.com/maxperutzlabs-ms/perutz-ms-contaminants).

Computational analysis was performed using Python and the in-house developed Python library MsReport (version 0.0.24, [Hollenstein et al. 2023]). LFQ protein intensities reported by FragPipe were log_2_-transformed and normalized across samples using the ModeNormalizer from MsReport. The missing normalized LFQ intensity values were imputed by drawing random values from a normal distribution after filtering out contaminants, proteins with less than two peptides and less than two quantified values in at least one group. Differences between groups were statistically evaluated using the LIMMA 3.52.1 (Ritchie et al. 2015) at 5% FDR (Benjamini-Hochberg). The in-house Python library XlsxReport (0.1.0) was used to create a formatted Excel file summarizing the results of protein quantification. Proteins were filtered to only include those that have at least six identified peptides and 24 spectral count events, both summed across all samples. Proteins were considered IGF2BP3 interactors if their normalized LFQ values were enriched more than four times over IgG control and had adjusted *P*-value of <0.05 for at least in one treatment condition. GO term analysis was performed with Enrichr webserver (Chen et al. 2013). Redundancy in GO terms was reduced using GOSemSim (Yu et al. 2010; Kuleshov et al. 2016) and rrvgo (Sayols 2023). Full GO term enrichment lists are in the Supplemental Material.

### Coimmunoprecipitation of endogenous IRE1

One 15 cm dish of 80% confluent HCT116 WT cells was used per condition. To induce ER stress, cells were treated with 5 µg/mL TM for 4 h, DMSO at a 1:1000 dilution was used as a control. Cells were washed with ice-cold PBS and collected by scraping and pelleting at 500*g* for 5 min at 4°C. Cells were lysed in 250 µL of lysis buffer (25 mM HEPES at pH 7.3, 150 mM NaCl, 0.5% NP-40, 0.5 mM EDTA, 10% glycerol, 1× EDTA-free protease inhibitor cocktail, 1× PhosSTOP) per condition by incubation for 15 min on ice with intermittent vortexing. The lysate was clarified by centrifugation at 20,000*g* for 20 min at 4°C. Ten micrograms of anti-IRE1 antibody (clone B-12, Santa Cruz Biotechnology sc-390960, lot J2521) were coupled to 40 µL of protein G Dynabeads (Invitrogen) in 1 mL of the lysis buffer for 20 min rotating at room temperature, washed three times with 1 mL of the lysis buffer, washed twice in 0.3 mL of the conjugation buffer (20 mM sodium phosphate, 150 mM NaCl at pH 7.5) and cross-linked to the Dynabeads with 5 mM bissulfosuccinimidyl suberate (BS3) in 1 mL of the conjugation buffer for 30 min rotating at room temperature. The reaction was quenched by addition of 25 µL of 1 m Tris-HCl (pH 7.5) followed by 15 min incubation rotating at room temperature. The Dynabeads with the cross-linked antibody were washed three times with 0.5 mL of the lysis buffer, resuspended in the original bead volume (40 µL) and added to 200 µL of the clarified lysate. The lysate was incubated with the beads for 3 h at 4°C. Beads were washed three times with 1 mL of the wash buffer (25 mM HEPES at pH 7.3, 150 mM NaCl, 0.5 mM EDTA, 10% glycerol). Each wash was incubated on ice for 3 min. For RNase digestion, 0.1 U/µL RNase I (Ambion) and 1 U/µL RNase T1 (EN0541) were added to the second wash. Samples were incubated at room temperature for 10 min on a rotator. Proteins were eluted in 25 µL of 1× SDS sample buffer for 5 min at +95°C, loaded on SDS-PAGE, and analyzed by Western blotting as described above (Supplemental Table S6).

### Coimmunoprecipitation of GFP-IGF2BP3 and mNeonGreen-EDC3

One 15 cm dish of 80% confluent HEK293T split GFP-IGF2BP3 or split mNeonGreen-EDC3 cells was used per condition. Parental HEK293T split GFP cells were used as a background control. To induce ER stress, cells were treated with 5 µg/mL TM for 4 h (if other time is not indicated), DMSO at a 1:1000 dilution was used as a control. Cells were washed with ice-cold PBS and collected by scraping and pelleting at 500*g* for 5 min at 4°C. Cells were lysed in 250 µL of lysis buffer (25 mM Tris-HCl at pH 7.4, 100 mM KCl, 0.5% NP-40, 10% glycerol, 1× EDTA-free protease inhibitor cocktail, 1 U/µL murine RNase inhibitor [NEB]) per condition by incubation for 10 min on ice. The lysate was clarified by centrifugation at 20,000*g* for 20 min at 4°C. The GFP-IGF2BP3 IP was performed using 40 µL of GFP-trap magnetic beads (ChromoTek) per condition. Twenty microliters of mNeonGreen-trap magnetic beads (ChromoTek) per condition were used for mNeonGreen-EDC3. The lysate was incubated with the beads for 1 h at 4°C with addition of 2 U/µL of RNase I (Ambion) and the beads were washed five times with 0.2 mL of ice-cold lysis buffer. Proteins were eluted in 25 µL of 1× SDS sample buffer for 5 min at +95°C, loaded on SDS-PAGE, and analyzed by Western blotting as described above with anti-IGF2BP3, anti-EDC4, and anti-EDC3 antibodies (Supplemental Table S6).

### Coimmunoprecipitation of GFP-EDC3 and 3xFLAG-IGF2BP3 WT and 4A mutant

One well of 6 well plate was used per condition. ∼70% confluent HCT116 tetON OsTIR1 IGF2BP3 KO cells clone A3 were transfected with transient expression plasmids expressing free eGFP (pEGFP-C1) or eGFP-EDC3 (pT7-EGFP-C1-HsEDC3) and 3xFLAG-hsIGF2BP3 WT or 4A mutant (this study). pT7-EGFP-C1-HsEDC3 was a gift from Elisa Izaurralde (Addgene plasmid 25032). Cells were transfected with a total of 1 µg of DNA with jetOptimus reagent (Polyplus) according to the manufacturer's instructions. Cells were washed with ice-cold PBS and lysed in 200 µL of lysis buffer (25 mM Tris-HCl at pH 7.4, 100 mM KCl, 0.5% NP-40, 10% glycerol, 1× EDTA-free protease inhibitor cocktail, 1 U/µL murine RNase inhibitor [NEB]) per condition by incubation for 10 min on ice. The lysate was clarified by centrifugation at 4°C 20,000*g* for 20 min. The lysate was incubated with the 20 µL GFP-trap beads for 1 h at 4°C with addition of 2 U/µL RNase I (Ambion) and the beads were washed five times with 0.2 mL of ice-cold lysis buffer. Proteins were eluted in 25 µL of 1× SDS sample buffer for 5 min at +95°C, loaded on SDS-PAGE and analyzed by Western blotting as described above with anti-GFP and anti-FLAG antibodies (Supplemental Table S6).

### In silico screening of IGF2BP3 protein–protein interactions

Protein–protein interaction screening of IGF2BP3 was performed using the AlphaFold2 Multimer (Jumper et al. 2021; Evans et al. 2022; Mirdita et al. 2022) via the HT-ColabFold pipeline (Hohmann et al. 2026). To this end pairwise interactions were analyzed for full length IGF2BP3 (O00425, IF2B3_HUMAN) versus IGF2BP3 interactors identified using co-IP–MS. In total 1213 predictions were made. PEAKscore was used to evaluate the prediction of the interaction. It represents the inverse, scaled (0–1) minimum precision alignment error (PAE) between analyzed chains. HT-ColabFold screening was done using the HPC of the Center for Integrative Bioinformatics Vienna (CIBIV) and Life Science Compute Cluster (LiSC) of the University of Vienna, Austria.

The interactions between the separate domains of IGF2BP3 and EDC3 were analyzed using AlphaFold 3 (Abramson et al. 2024). The domain separation was defined using the following boundaries: IGF2BP3 (O00425, IF2B3_HUMAN) RRM1/2—amino acids 1–195, linker1-KH1/2—amino acids 160–350, KH1/2-linker2—amino acids 196–405, linker2-KH3/4—amino acids 343–579; EDC3 (Q96F86, EDC3_HUMAN) SM—amino acids 1–68, YjeF—amino acids 283–487.

### IGF2BP3 protein expression and purification

Full-length IGF2BP3 WT and 4A constructs were cloned into a pGEX-6P-2 vector containing an N-terminal GST-tag and a 3C cleavage site. Protein purification was performed as described by Hornegger et al. (2024). Specifically, the proteins were expressed in Rosetta (DE3) cells grown to an OD of ∼0.7 and induced with 400 µM IPTG. Cells were grown overnight at 20°C, pelleted and frozen in liquid nitrogen. Proteins were purified by resuspending the pellet in GST lysis buffer (25 mM HEPES at pH 7.2, 1 M NaCl, 5% glycerol, 2 mM DTT, 0.5 mM PMSF, 2 mM EDTA) and lysing the cells in a EmulsiFlex C3 homogenizer. After pelleting nonsoluble cell debris was removed by centrifugating the lysate at 40,000*g* for 30 min at 4°C. Proteins were loaded onto a GST HiTrap HP 5 mL column, washed with GST wash buffer (25 mM HEPES at pH 7.2, 500 mM NaCl, 5% glycerol, 2 mM DTT, 0.5 mM PMSF, 2 mM EDTA) and eluted with a gradient of 20 mM reduced glutathione in GST wash buffer. Bound nucleotides were removed by loading the proteins onto a Heparin HiTrap HP 5 mL column after diluting the NaCl concentration to 100 mM (with wash buffer without NaCl), washing them with wash buffer (25 mM HEPES at pH 7.2, 100 mM NaCl, 5% glycerol, 2 mM DTT, 0.5 mM PMSF, 2 mM EDTA) and eluting them with a gradient of 1 M NaCl in wash buffer. The GST-tag was cleaved off by incubating the eluted protein with GST-3C overnight at 4°C. The tag and the 3C protease were separated by running the cleaved protein over a GST HiTrap 5 mL column using the same buffers as during the affinity purification. The flowthrough was pooled and further purified via size exclusion chromatography (SEC). Proteins were eluted with SEC buffer (25 mM HEPES, 150 mM NaCl, 2 mM DTT). Protein concentration was measured using a NanoDrop.

### Analytical size exclusion chromatography

Purified IGF2BP3 WT and IGF2BP3 4A proteins were diluted to 20 µM in SEC buffer (25 mM HEPES at pH 7.2, 150 mM NaCl, 2 mM DTT) and centrifuged at 14,000*g* for 10 min at 4°C to remove insoluble aggregates. Twenty microliters of each sample was injected onto a Superdex 200 Increase 5/150 GL column (Cytiva) equilibrated in Size Exclusion Chromatography (SEC) buffer and connected to an ÄKTA pure micro chromatography system (Cytiva) at 4°C. Proteins were separated at a flow rate of 0.1 mL/min. Elution was monitored by UV absorbance at 280 nm. Fractions were collected in 50 µL increments. Fractions corresponding to elution volumes between 1.4 and 1.8 mL, spanning the main absorbance peak at 280 nm, were analyzed by SDS-PAGE using self-cast 12% acrylamide gels under denaturing and reducing conditions. Electrophoresis was performed at 200 V for 45 min. Proteins were visualized by Coomassie staining.

### Circular dichroism

Purified IGF2BP3 WT and IGF2BP3 4A were thawed, centrifuged at 20,000*g* for 15 min at 4°C and dialyzed into 1 L of circular dichroism buffer [25 mM potassium phosphate, 150 mM (NH_4_)_2_ SO_4_ at pH 7.3] overnight at 4°C using 3.5 kDa MWCO dialysis tubing (Thermo Fisher lot X352983). Next day, proteins were centrifuged at 20,000*g* for 15 min at 4°C and diluted to 0.2 mg/mL with circular dichroism buffer. The CD spectral measurement was performed at 20°C in a 1 mm pathlength quartz cuvette using a Chirascan Plus CD spectrometer (Applied Photophysics) in triplicates. The spectrometer was purged with nitrogen overnight. The purge check and the absolute lower wavelength and high voltage signal quality checks were performed preceding the sample measurement (1 nm bandwidth, 0.5 step size, 0.5 sec integration time). The circular dichroism buffer background spectrum was acquired and subtracted prior to spectral analysis in GraphPad Prism (192–260 nm). For each spectrum, normalization was performed using the minimum and maximum intensity values measured within the 192–260 nm wavelength range as reference points. The circular dichroism spectroscopy measurement was performed at the Protein Technologies Facility at Vienna BioCenter Core Facilities (VBCF), member of the Vienna BioCenter (VBC), Austria.

### Fluorescence anisotropy

Fluorescence polarization assays were performed between IGF2BP3 WT and 5′-fluorescein-labeled RNA probes (5′FluorT-AAUCCAGAA, 5′FluorT-AACAUUUAA, 5′FluorT-AAACUGUAA, and 5′FluorT-AAAAAAAAA; synthesized and RNase-free HPLC purified by Integrated DNA Technologies Germany GmbH) as follows: IGF2BP3 WT was thawed and centrifuged at 20,000*g* for 15 min at 4°C. The sample with the highest protein concentration was prepared in anisotropy buffer (25 mM HEPES at pH 7.2, 150 mM NaCl) in a 384 well plate (Corning). A concentration series was created by diluting the sample 1:1 with anisotropy buffer. Final protein concentrations ranged from 3.9 nM to 4 µM. One microliter of 10 × RNA containing buffer (250 nM RNA, 25 mM HEPES, 150 mM NaCl at pH 7.2) was added per well to reach a final concentration of 25 nM of fluorescein-labeled RNA in 10 µL reaction volume. Subsequently, samples were incubated for 30 min on ice. The fluorescence anisotropy was measured in triplicates using a Tecan Sparc spectrofluorometer at 525 nm with a bandwidth of 20 nm at 20°C and an excitation wavelength of 485 nm, a band width of 10 nm, 30 flashes with gain 75, automatic mirror setting, 0 sec settling time and *Z*-position at 19,300 µm. The G-factor was determined once for every experiment and used to calculate the anisotropy by measuring 25 nM RNA in anisotropy buffer in the beginning of the experiment. The fluorescence anisotropy value was calculated by the manufacturer's software. KDs were calculated in GraphPad Prism by using a dose response curve to fit the data using following model: *Y* = bottom + (top − bottom)/1 + 10^[log10(*K*d) − log10(*x*)] × *n*^.

### Data deposition

Next-generation sequencing data have been deposited in GEO with the following identifiers: GSE289214 (RNA-seq of HCT116 cells upon IGF2BP3 CRISPR/Cas9 knockout in control and ER stress conditions), GSE289023 (IR-PAR-CLIP of IGF2BP3 in HCT116 cells in control and ER stress conditions), GSE289024 (transcriptome [QuantSeq] of HCT116 cells in control and ER stress conditions matching IR-PAR-CLIP samples), GSE289424 (transcriptome [RNA-seq] of HCT116 cells upon IGF2BP3 siRNA knockdown in control and ER stress conditions), GSE289482 (SLAMseq of HCT116 cells upon IGF2BP3 knockdown in control and endoplasmic reticulum [ER] stress conditions), and GSE289480 (SLAMseq of doxycycline-inducible knockout and siRNA-mediated depletion of IGF2BP3 in RKO iCas9 cells). Mass spectrometry proteomics data have been deposited at the ProteomeXchange Consortium via the PRIDE partner repository (Perez-Riverol et al. 2022) with the data set identifiers PXD073640 (total proteome of IGF2BP3 CRISPR/Cas9 knockout cells in control and ER stress conditions) and PXD060548 (coimmunoprecipitation of endogenous IGF2BP3 followed by mass spectrometry in control and ER stress conditions).

## Supplemental Material

Supplement 1
