# Supplementary material for: Dual regulation of the unfolded protein response by IGF2BP3 during ER stress
Source: Genes Dev. 2026 Aug 1;40(15-16):1304–29. doi: 10.1101/gad.353291.125 (PMC13431160; doi:10.1101/gad.353291.125)
Supplement: Supplement 1 [file Supplemental_Data.pdf]

Supplemental Data

Dual regulation of the unfolded protein response by IGF2BP3 during ER stress

Aleksandra S Anisimova[1,2], Sabina Omerbegovic [1,5], Milica Mihailovic [1,2], Sascha Gratzl [1,2], Harald Hornegger[1,2], Irmgard Fischer[1], Gijs A Versteeg[1,3], Stefan L Ameres[1,4], G Elif Karagöz[1,5,#]

1. Max Perutz Labs, Vienna BioCenter Campus, Dr.-Bohr-Gasse 9, 1030, Vienna, Austria.
2. Vienna BioCenter PhD Program, a Doctoral School of the University of Vienna and the Medical University of Vienna, A-1030 Vienna, Austria.
3. University of Vienna, Center for Molecular Biology, Department of Microbiology, Immunobiology, and Genetics, Dr.-Bohr-Gasse 9, 1030, Vienna, Austria.
4. University of Vienna, Center for Molecular Biology, Department of Biochemistry and Cell Biology, Dr.-Bohr-Gasse 9, 1030, Vienna, Austria.
5. Medical University of Vienna, Center for Medical Biochemistry, Dr.-Bohr-Gasse 9, 1030, Vienna, Austria.

This file contains **Supplemental Figures S1 – S7**, **Supplemental References**.

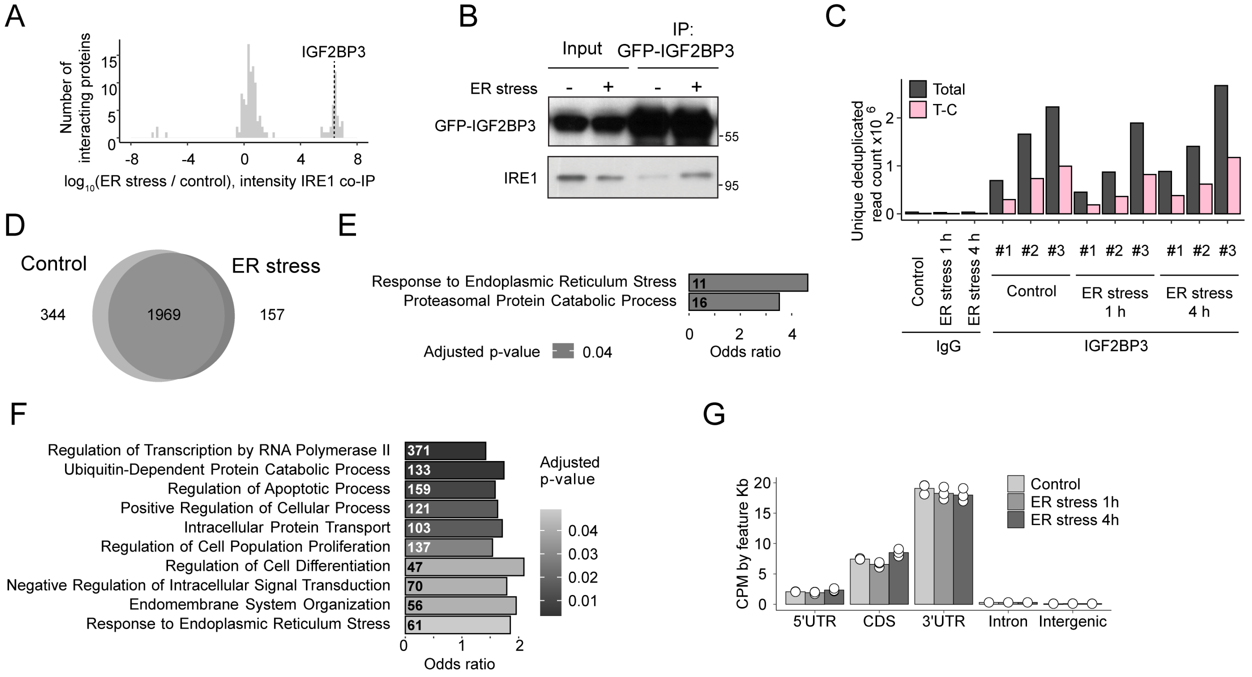

Supplemental Figure S1. A. Histogram showing the distribution of fold changes in normalized peptide intensity values of IRE1 interaction partners upon ER stress (crosslinking mass spectrometry data from Acosta-Alvear, Karagöz et al., 2018 (Acosta-Alvear, Karagoz et al. 2018)). Proteins identified in IgG or non-crosslinking controls were excluded from the analysis (n = 126). **B.** Western blot of IRE1 showing its association with IGF2BP3 after immunoprecipitation of GFP-IGF2BP3 from HEK293T cells treated with ER stress-inducing drug TM at 5 µg/mL for 4 hours. **C.** Barplots showing numbers of total and T-C containing unique deduplicated IR-PAR-CLIP read counts per library before normalization. **D.** Venn diagram showing the intersection of IGF2BP3 IR-PAR-CLIP targets in DMSO control and ER stress conditions (4-hour treatment with 5 µg/mL tunicamycin). **E.** GO term analysis of 157 IGF2BP3-bound transcripts identified in only in ER stress conditions **F.** GO term analysis of 2470 IGF2BP3-bound transcripts identified in either control or ER stress conditions. **G.** Feature length-normalized aggregated IGF2BP3 IR-PAR-CLIP coverage of genomic features. For IR-PAR-CLIP and transcriptome experiments n=3 biological replicates.

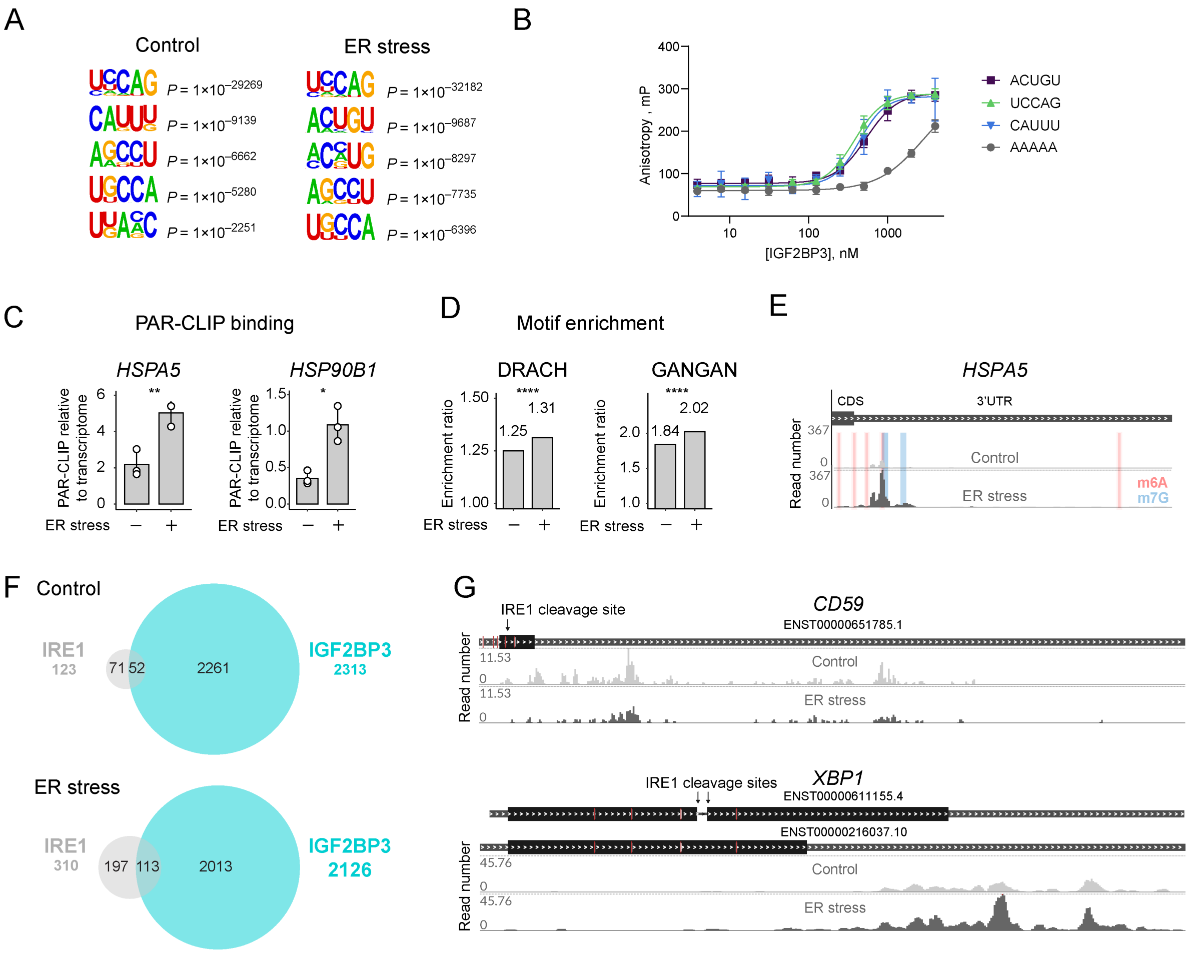

Supplemental Figure S2. A. 5nt-long consensus motifs enriched in IGF2BP3 IR-PAR-CLIP reads over scrambled control in control and ER stress conditions (4 hr). Motif enrichment analysis was performed using HOMER software on pooled replicates. **B.** Fluorescence anisotropy experiments of full-length IGF2BP3 WT and 5′-fluorescein-labeled RNA probes containing the indicated motif. Data are the mean ± s.d of n=3 technical replicates. The curves represent the fit of the Hill equation (see Materials and Methods). **C.** IGF2BP3 binding values (IR-PAR-CLIP CPM / QuantSeq CPM) for selected genes. Data are the mean ± s.d of n=3 biological replicates. *P* values were calculated by unpaired two-sided Student's t-test. **D.** Motif enrichment analysis of IGF2BP3 IR-PAR-CLIP reads for m6A consensus motif DRACH and m7G consensus motif GANGAN. First, the motif enrichment was calculated for the DMSO control samples over scrambled background. Next, the motif enrichment was calculated for the samples treated with TM for 4 hours using DMSO control sample as a background. Analysis was performed using MEME suite SEA software on pooled replicates. **E.** Representative IGF2BP3 IR-PAR-CLIP coverage example for 3' UTR of *HSPA5.* m6A ((Liang, Ye et al. 2024), see Materials and Methods) and m7G (Malbec, Zhang et al. 2019) methylation sites are highlighted. **F.** Venn diagram showing the intersection between IGF2BP3 and IRE1 PAR-CLIP targets (Acosta-Alvear, Karagoz et al. 2018) in control and ER stress conditions. **G.** Representative IGF2BP3 IR-PAR-CLIP coverage of IRE1 targets *XBP1* and *CD59*. $^{*}P < 0.05$; $^{**}P < 0.01$; $^{***}P < 0.001$; $^{****}P < 0.0001$.

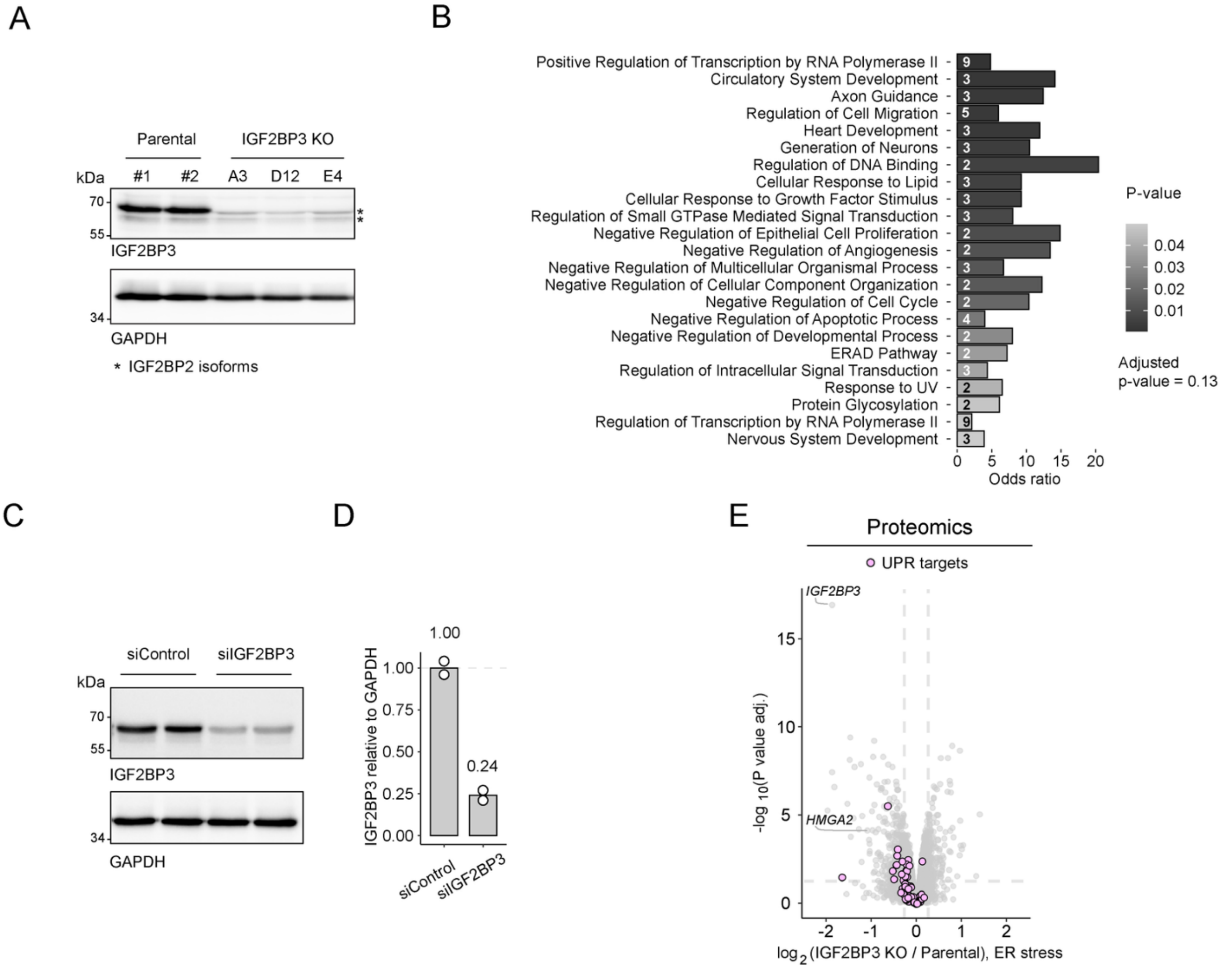

Supplemental Figure S3. A. Western blot of parental and IGF2BP3 KO HCT116 cell lines (clones A3, D12 and E4). * IGF2BP2 isoforms recognized by polyclonal anti-IGF2BP3 antibody. **B.** GO term analysis of genes downregulated in IGF2BP3 KO comparing to parental cells during ER stress. edgeR glmQLFTest *P* value adj. < 0.05. **C.** Western blot showing depletion of IGF2BP3 after 48-hour treatment with siRNA against IGF2BP3 compared to control siRNA. **D.** Quantification of C. n=2 technical replicates. **E.** Volcano plot of total proteome changes upon IGF2BP3 KO in ER stress conditions. n=4 biological replicates. *P* values were calculated using limma-trend moderated t-test.

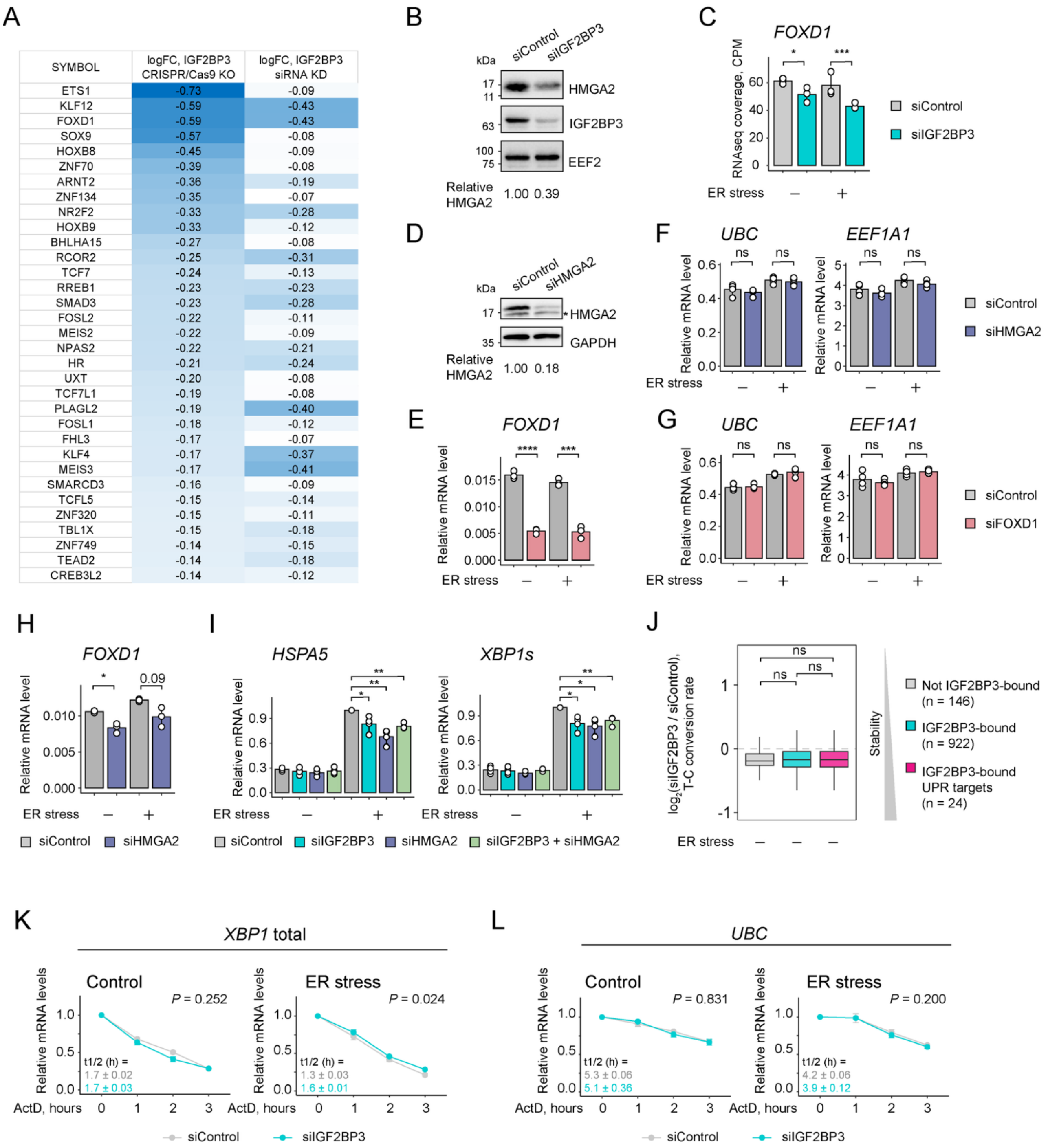

SYMBOL	logFC, IGF2BP3 CRISPR/Cas9 KO	logFC, IGF2BP3 siRNA KD
ETS1	-0.73	-0.09
KLF12	-0.59	-0.43
FOXD1	-0.59	-0.43
SOX9	-0.57	-0.08
HOXB8	-0.45	-0.09
ZNF70	-0.39	-0.08
ARNT2	-0.36	-0.19
ZNF134	-0.35	-0.07
NR2F2	-0.33	-0.28
HOXB9	-0.33	-0.12
BHLHA15	-0.27	-0.08
RCOR2	-0.25	-0.31
TCF7	-0.24	-0.13
RREB1	-0.23	-0.23
SMAD3	-0.23	-0.28
FOSL2	-0.22	-0.11
MEIS2	-0.22	-0.09
NPAS2	-0.22	-0.21
HR	-0.21	-0.24
UXT	-0.20	-0.08
TCF7L1	-0.19	-0.08
PLAGL2	-0.19	-0.40
FOSL1	-0.18	-0.12
FHL3	-0.17	-0.07
KLF4	-0.17	-0.37
MEIS3	-0.17	-0.41
SMARCD3	-0.16	-0.09
TCFL5	-0.15	-0.14
ZNF320	-0.15	-0.11
TBL1X	-0.15	-0.18
ZNF749	-0.14	-0.15
TEAD2	-0.14	-0.18
CREB3L2	-0.14	-0.12

Supplemental Figure S4. **A.** Table showing log_2(IGF2BP3 depletion / control) values for transcriptional regulators (GO:0140110) that were downregulated more than 10% or 5% upon IGF2BP3 CRISPR/Cas9- or siRNA-mediated depletion, respectively, under ER stress conditions. **B.** Western blot showing HMGA2 levels upon siRNA-mediated depletion of IGF2BP3. **C.** FOXD1 transcript levels (RNA-Seq, CPM) upon siRNA-mediated depletion of IGF2BP3. Data are the mean ± s.d. of n=3 biological replicates. *P* values were calculated by edgeR glmQLFTest. **D.** Western blot confirming siRNA-mediated depletion of HMGA2 * unspecific band. **E.** RT-qPCR

confirming siRNA-mediated depletion of *FOXD1*. **F** and **G.** RT-qPCR of control transcripts *UBC* and *EEF1A* upon siRNA-mediated depletion of HMGA2 and FOXD1, respectively. **H.** RT-qPCR of *FOXD1* upon siRNA-mediated depletion of HMGA2. **I.** RT-qPCR comparing levels of *HSPA5*, *XBP1* spliced, and *ATF4* upon siRNA-mediated depletion of IGF2BP3, HMGA2, or combined depletion of both. **J**. Boxplot showing changes in estimated transcript stability (T-C conversion rates) in unstressed conditions following siRNA-mediated IGF2BP3 depletion for IGF2BP3-bound transcripts, IGF2BP3-bound UPR targets, and non-IGF2BP3 bound transcripts (IR-PAR-CLIP CPM / QuantSeq CPM < 0.1). *P* values were calculated by two-sided Wilcoxon test. **K** and **L.** RT-qPCR analyses of degradation rates of the UPR target *XBP1* (total) and the control transcript *UBC* upon siRNA-mediated depletion of IGF2BP3. HCT116 cells were treated with TM at 250 ng/mL for 4 hours and the transcription was blocked with 5 µg/mL ActD for indicated time points. Data are the mean ± s.e. of n=4 biological replicates. *P* values were calculated by paired two-sided Student's t-test. For the SLAMseq experiments n=4 biological replicates. In RT-qPCR analyses, mRNA levels were normalized to *RPL6*. ER stress was induced with 100 ng/mL TM for 8 hours. **P* < 0.05; ***P* < 0.01; ****P* < 0.001; *****P* < 0.0001.

A

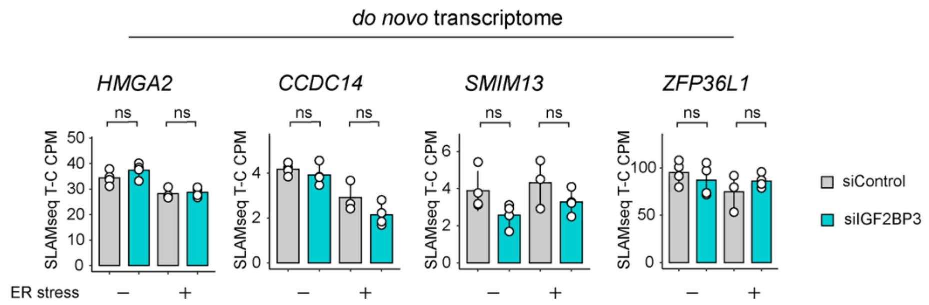

Supplemental Figure S5. A.Barplots showing SLAMseq *de novo* (T-C CPM) transcript levels for selected transcripts regulated by IGF2BP3. Data are the mean ± s.d of n=4 biological replicates. *P* values were calculated by unpaired two-sided Student's t-test.

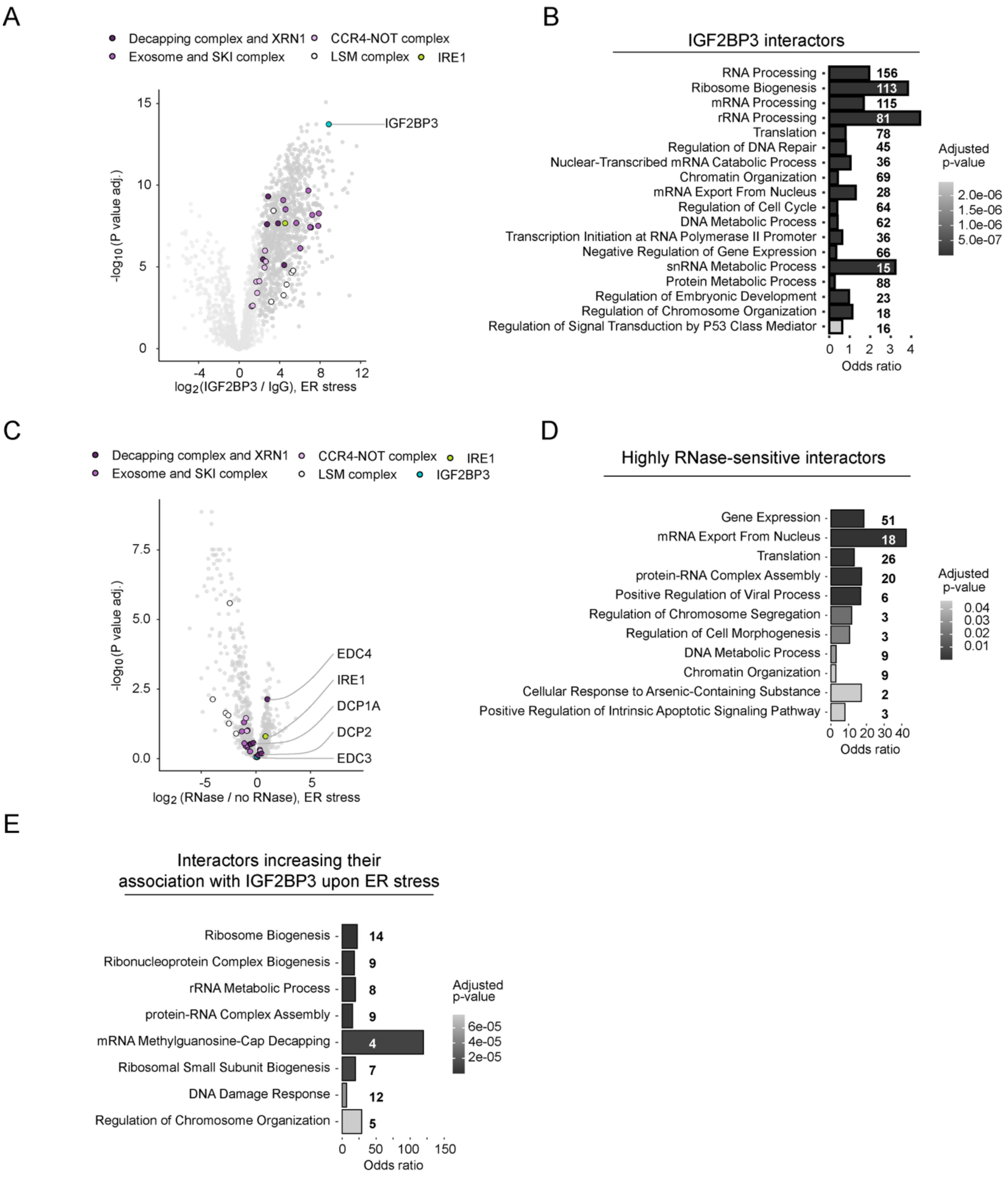

Supplemental Figure S6. **A.** Volcano plot of co-IP–MS analysis of endogenous IGF2BP3 upon ER stress (TM, 5 µg/mL, 4 h) using IGF2BP3 antibody versus IgG control. IGF2BP3 interaction partners (4 times enriched over IgG control, *P* value adj. < 0.05) are shown in dark gray. **B.** GO term enrichment analysis of IGF2BP3 interaction partners (4 times enriched over IgG control at least in one treatment condition, *P* value adj. < 0.05). **C.** Volcano plots comparing the IGF2BP3 interactome with and without RNase treatment. **D.** GO term enrichment analysis of IGF2BP3-

interacting proteins that decreased their association with IGF2BP3 upon RNase treatment (*P* value adj. < 0.05). **E.** GO term enrichment analysis of IGF2BP3-interacting proteins that increased their association with IGF2BP3 upon ER stress for more than 20%. For IGF2BP3 co-IP–MS analysis n=3 biological replicates.

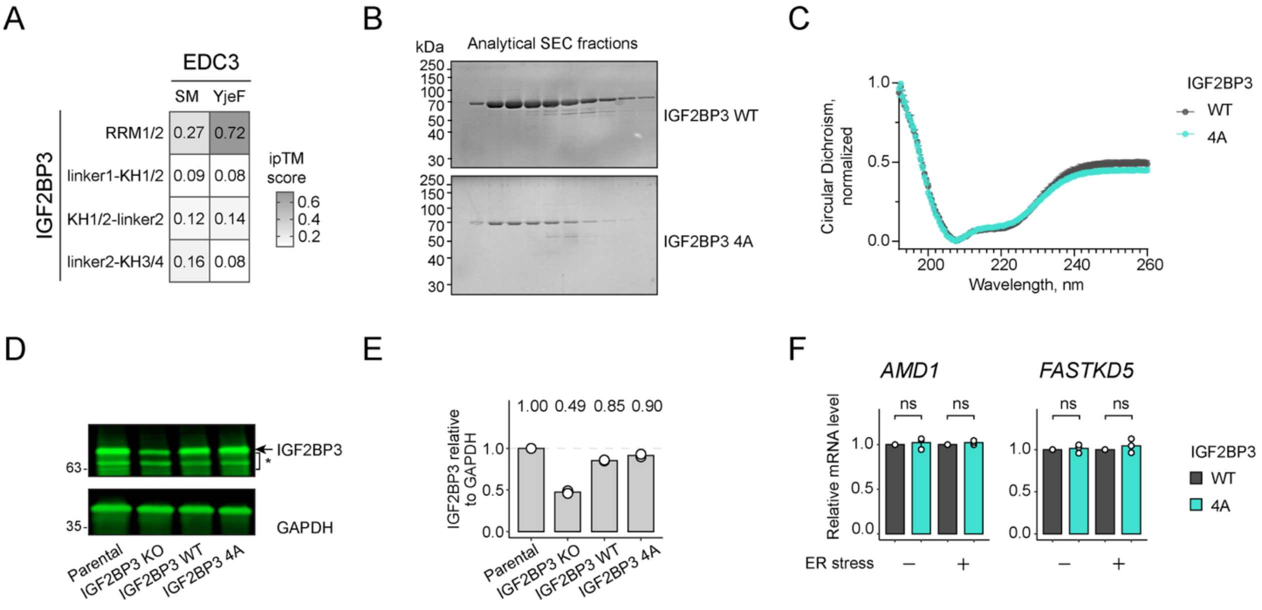

Supplemental Figure S7. A. Matrix showing AlphaFold3 ipTM scores between separate domains of IGF2BP3 and EDC3. **B.** Coomassie-stained SDS-PAGE of fractions from analytical size-exclusion chromatography (SEC) of IGF2BP3 WT and the IGF2BP3 4A mutant. **C.** Circular dichroism spectra of IGF2BP3 WT and the IGF2BP3 4A mutant. Data are the mean ± s.d. of n=3 technical replicates. **D.** Near-infrared Western blot showing expression levels of IGF2BP3 WT and the IGF2BP3 4A mutant introduced into IGF2BP3 KO HCT116 cell line. * IGF2BP2 isoforms recognized by the polyclonal anti-IGF2BP3 antibody. **E.** Quantification of D. Data are the mean ± s.d. of n=2 technical replicates. **F.** RT-qPCR of control mRNAs in HCT116 cells expressing IGF2BP3 WT or IGF2BP3-4A mutant. mRNA levels were normalized to *RPL6* and shown relative to IGF2BP3 WT. Data are the mean ± s.d. of n=3 biological replicates. *P* values were calculated by unpaired two-sided Student's t-test. ER stress was induced with 5 µg/mL TM for 4 hours.

Supplemental references

Acosta-Alvear, D., G. E. Karagoz, F. Frohlich, H. Li, T. C. Walther and P. Walter (2018). "The unfolded protein response and endoplasmic reticulum protein targeting machineries converge on the stress sensor IRE1." Elife **7**.

Liang, Z., H. Ye, J. Ma, Z. Wei, Y. Wang, Y. Zhang, D. Huang, B. Song, J. Meng, D. J. Rigden and K. Chen (2024). "m6A-Atlas v2.0: updated resources for unraveling the N6-methyladenosine (m6A) epitranscriptome among multiple species." Nucleic Acids Res **52**(D1): D194-D202.

Malbec, L., T. Zhang, Y. S. Chen, Y. Zhang, B. F. Sun, B. Y. Shi, Y. L. Zhao, Y. Yang and Y. G. Yang (2019). "Dynamic methylome of internal mRNA N(7)-methylguanosine and its regulatory role in translation." Cell Res **29**(11): 927-941.
